# Assignment of Atlantic salmon (*Salmo salar) *linkage groups to specific chromosomes: Conservation of large syntenic blocks corresponding to whole chromosome arms in rainbow trout (*Oncorhynchus mykiss*)

**DOI:** 10.1186/1471-2156-10-46

**Published:** 2009-08-18

**Authors:** Ruth B Phillips, Kimberly A Keatley, Matthew R Morasch, Abigail B Ventura, Krzysztof P Lubieniecki, Ben F Koop, Roy G Danzmann, William S Davidson

**Affiliations:** 1 Department of Biological Sciences, Washington State University, Vancouver, WA 98686-9600, USA; 2 Center for Reproductive Biology, Washington State University, Pullman, WA 99164-4236, USA; 3 Department of Molecular Biology and Biochemistry, Simon Fraser University, Burnaby, British Columbia V5A 1S6, Canada; 4 Department of Biology, University of Victoria, Victoria, British Columbia V8W 3P2, Canada; 5 Department of Integrative Biology, University of Guelph, Guelph, Ontario N1G 2W1, Canada

## Abstract

**Background:**

Most teleost species, especially freshwater groups such as the Esocidae which are the closest relatives of salmonids, have a karyotype comprising 25 pairs of acrocentric chromosomes and 48–52 chromosome arms. After the common ancestor of salmonids underwent a whole genome duplication, its karyotype would have 100 chromosome arms, and this is reflected in the modal range of 96–104 seen in extant salmonids (e.g., rainbow trout). The Atlantic salmon is an exception among the salmonids as it has 72–74 chromosome arms and its karyotype includes 12 pairs of large acrocentric chromosomes, which appear to be the result of tandem fusions. The purpose of this study was to integrate the Atlantic salmon's linkage map and karyotype and to compare the chromosome map with that of rainbow trout.

**Results:**

The Atlantic salmon genetic linkage groups were assigned to specific chromosomes in the European subspecies using fluorescence *in situ *hybridization with BAC probes containing genetic markers mapped to each linkage group. The genetic linkage groups were larger for metacentric chromosomes compared to acrocentric chromosomes of similar size. Comparison of the Atlantic salmon chromosome map with that of rainbow trout provides strong evidence for conservation of large syntenic blocks in these species, corresponding to entire chromosome arms in the rainbow trout.

**Conclusion:**

It had been suggested that some of the large acrocentric chromosomes in Atlantic salmon are the result of tandem fusions, and that the small blocks of repetitive DNA in the middle of the arms represent the sites of chromosome fusions. The finding that the chromosomal regions on either side of the blocks of repetitive DNA within the larger acrocentric chromosomes correspond to different rainbow trout chromosome arms provides support for this hypothesis.

## Background

The common ancestor of salmonid fishes, which include Atlantic salmon and rainbow trout, underwent a whole genome duplication sometime within the early Tertiary to the late Cretaceous periods [1]. The evidence for a salmonid-specific auto-tetraploidization comes in part from the size of their genomes, their chromosomal organization, the presence of a large number of duplicate genes and residual tetrasomic inheritance in some species [2]. Salmonids have about twice as much DNA per cell as their closest relatives, the Esociformes and Osmeriformes [3]. Most salmonids have karyotypes composed of both metacentric (bi-armed) and acrocentric (uni-armed) chromosomes. The modal range of chromosome arms (NF) in salmonids is 96–104 [4] whereas most teleost species, especially freshwater groups such as the Esocidae, have a karyotype with 25 pairs of acrocentric chromosomes and NFs of 48–52 [5].

The karyotype of Atlantic salmon is the exception among the salmonids as it has an NF of 72–74 and includes 12 large acrocentric chromosome pairs, which appear to be the result of tandem fusions [4]. Substantial intra-specific variation in the karyotype of Atlantic salmon has been documented with the North American subspecies having 2N = 54 (NF = 72) and the European subspecies usually having 2N = 58 (NF = 74) [4]. Examination of these two karyotypes suggests that up to 8 chromosomal rearrangements have occurred between these two subspecies [6]. The smallest metacentric chromosome in the European subspecies of Atlantic salmon has a short arm, which is very bright with DAPI staining [6] and is comprised entirely of 5S rDNA and the major ribosomal DNA locus [7].

Genetic linkage maps have been constructed for European Atlantic salmon using microsatellites [8], AFLPs [9] and SNPs [10]. Many of the markers from these maps have been integrated into a map that contains ~1,500 markers [11]. More than half of the microsatellite markers in the latter linkage map come from BAC end sequences. This has allowed the integration of the linkage map and the physical map [12], which is based on HindIII fingerprinting of Bacterial Artificial Chromosomes clones (BACs) from a BAC library (CHORI-214) constructed from the DNA of a single Norwegian male salmon from an aquaculture stock [13] (see ). Cytogenetic mapping utilizing fluorescence *in situ *hybridization (FISH) with BAC probes containing microsatellite loci that are linked to the male phenotype in Atlantic salmon identified the sex chromosome pair as chromosome 2 [14]. This also provided the first integration of the linkage map and the karyotype by positioning genetic markers on linkage group (LG) 1 on chromosome 2. Here we report the assignment and orientation of linkage groups to each chromosome pair in the European subspecies of Atlantic salmon. We then compared the resulting Atlantic salmon chromosome map with that of rainbow trout [15].

## Results

**Figure 1:**
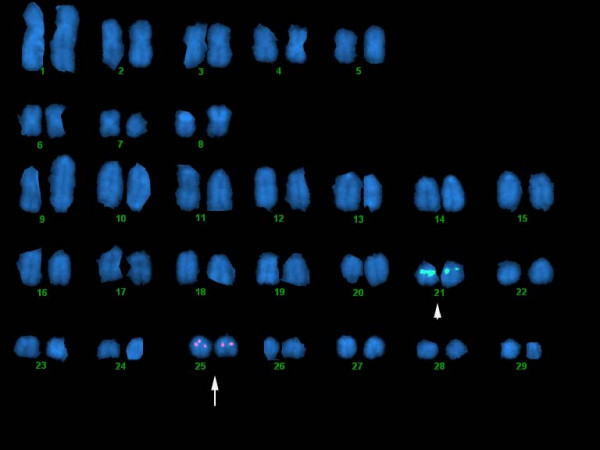
**Atlantic salmon chromosomes showing a dual hybridization with a BAC clone (S0004J13 labeled in red) containing myostatin-1 and Ssa0283BSFU (mapped to LG 20), which hybridizes to Ssa25, and a second BAC clone (S0046P15 labeled in green) containing myostatin-2 and Ssa0402BSFU (mapped to LG 14), which hybridizes to Ssa21**.

**Figure 2:**
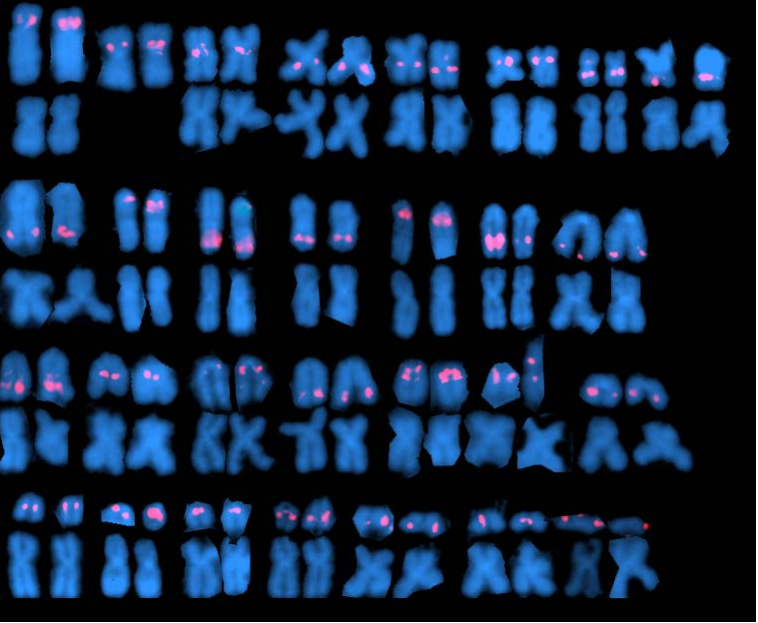
**Composite of 29 partial Atlantic salmon karyotypes showing results of hybridization with BAC clones containing markers mapped to each linkage group**. In each case, the sex chromosome pair from the same metaphase cell is shown below the chromosome pair containing the probe signal to indicate relative size, except for the sex chromosome pair itself, which is the second largest metacentric chromosome. Probes shown are named according to the marker that the BAC contains-followed by the BAC clone: Ssa1: BHMS281-S0198E23, Ssa2 (sex chromosome): Oneu18ASC-S0119E21, Ssa3: Ssa86-S0034P12, Ssa4: Ssa0133BSFU-S0426K01, Ssa5: BHMS206-S0026A22, Ssa6: Ssa0043BSFU-S0322O19, Ssa7: Ssa0037BSFU-S0121A15, Ssa8: Ssa0007BSFU-S0214J02, Ssa9: SSOSL85-S0040B06, Ssa10: Ssa0020BSFU-S0439A22, Ssa11: Ssa0982BSFU-S0056B24, Ssa12: BHMS146-S0195D10, Ssa13: Ssa0008BSFU-S0229A14, Ssa14: Ssa1027BSFU-S0008I14, Ssa15: Ssa197DU-S0121A09, Ssa16: OMM1013-S0097E23, Ssa17: BHMS304-S0031H11, Ssa18: Ssa0125BSFU-S0036I05, Ssa19: BHMS289-S0006P09, Ssa20: Ssa0730BSFU-S0067N15, Ssa21: Ssa0402BSFU-S0259B12, Ssa22: Ssa65-S0018L24, Ssa23: BHMS377-S0102N22, Ssa24: Ssa0006BSFU-S0191E15, Ssa25: BHMS241-S0014L21, Ssa26: Ssa0800BSFU-S0059P02, Ssa27: BHMS127-S0166I14, Ssa28: Ssa224-S0021G10, Ssa29: Ssa0084BSFU-S0016D16.

Hybridization experiments were carried out with BAC clones containing genetic markers mapped to each linkage group. It should be noted that there are 29 linkage groups in the Atlantic salmon genetic map. The nomenclature for the linkage groups is historical, and linkage groups 26, 27, 29 and 30 no longer exist (i.e., they were recognized as parts of larger linkage groups as more mapping data were added). With the exception of two very small chromosome pairs, multiple BAC clones were assigned to each chromosome pair. Dual hybridizations were done to confirm assignment of probes from several pairs of duplicate genes (lactate dehydrogenase A1 and lactate dehydrogenase A2, metallothionien A and metallothionien B, MHCIA and MHCIB and myostatin-1 and myostatin-2). Figure 1 shows a dual hybridization with BAC clone S0004J13 (labeled in red), which contains microsatellite Ssa0283BSFU and myostatin-1, and BAC clone S0046P15 (labeled in green), which contains microsatellite Ssa0402BSFU and myostatin-2. S0046P15 hybridizes to chromosome 21 (LG 14) and S0004J13 to chromosome 25 (LG 20). A composite of images showing the results of hybridizations of one probe for each linkage group is shown in Figure 2. In each case, the sex chromosome pair from the same metaphase spread is shown below the chromosome containing the probe signal to indicate its relative size. The sex chromosome is the second largest in the karyotype and has a telomeric band of repetitive DNA that is visible with DAPI staining [14].

**Figure 3:**
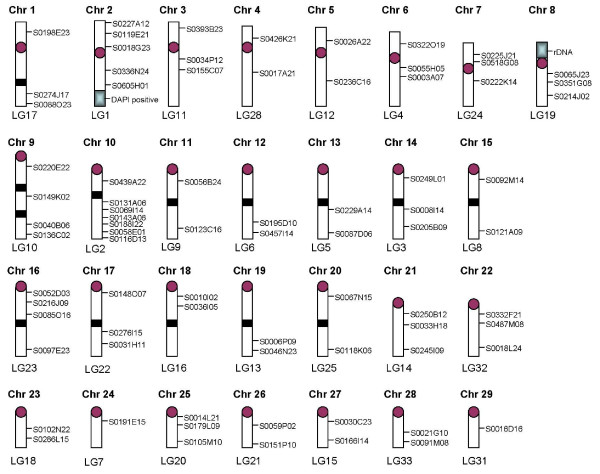
**Ideogram of the European Atlantic salmon karyotype showing the location of the BAC probes mapped by FISH**. Information concerning these BACs and their corresponding genetic markers is given in Additional File 1.

The BAC clones used to assign the genetic linkage groups to chromosomes are given in Additional File 1. An ideogram of the European Atlantic salmon karyotype showing the location of the BAC probes used to assign linkage groups to chromosomes is shown in Figure 3. Note that the sections of the large acrocentric chromosomes proximal and distal to the central block of repetitive DNA are labeled qa and qb, respectively. The largest acrocentric chromosome pair has two blocks of repetitive DNA dividing the arm into three parts: 9qa, 9qb and 9qc.

Table 1 shows the relative sizes (in cM) of the genetic linkage groups from the consensus female Atlantic salmon map [11], which has been updated in this study with the addition of 153 new markers, as well as the rank order with respect to size for the Atlantic salmon chromosomes. For both metacentric and acrocentric chromosome pairs there is generally a good correlation between chromosome size and size of the linkage group, but metacentric chromosomes of the same size had significantly larger linkage maps than acrocentric chromosome pairs. For example, the two largest chromosomes in the karyotype are a metacentric chromosome (#1 in Figure 3) and an acrocentric chromosome (#9 in Figure 3), but the linkage group sizes are 174 cM for LG 17, which corresponds to the largest metacentric chromosome, and 105 cM for LG 10, which corresponds to the largest acrocentric chromosome.

**Table 1:** Atlantic salmon chromosomes and linkage group rankings according to size.

**Chromosome**	**LG**	**LG Size in cM**	**Rank Chromosome Size**
1 M*	17	174.2	1
2 M	1 (sex)	110.7	5
3 M	11	104.2	8
4 M	28	91.1	9
5 M	12	104.3	10
6 M	4	100.9	14
7 M	24	104.2	18
8 M	19	78.1	21
9 A	10	105.3	2
10 A	2	95.5	3
11 A	9	86.9	4
12 A	6	77.1	6
13 A	5	90.1	7
14 A	3	53.1	29
15 A	8	65.1	11
16 A	23	97.7	12
17 A	22	92.5	13
18 A	16	95.5	15
19 A	13	72.7	16
20 A	25	77.0	17
21 A	14	60.8	19
22 A	32	70.5	20
23 A	18	55.3	22
24 A	7	56.5	23
25 A	20	71.6	24
26 A	21	35.8	25
27 A	15	37.9	26
28 A	33	50.0	27
29 A	31	57.5	28

*M indicates a metacentric chromosome and A indicates an acroentric chromosome.

Table 2 shows the comparison of the genetic and cytogenetic maps for Atlantic salmon compared to rainbow trout. Large blocks corresponding to whole chromosome arms in rainbow trout correspond to chromosome arms or qa and qb portions of the large acrocentric chromosome pairs in Atlantic salmon. Although there is extensive conservation of synteny in these regions, some rearrangements appear to have occurred between the species such that the orders of markers along these chromosomal segments differ in these species. As the densities of markers in the Atlantic salmon and rainbow trout linkage maps increase, the confidence in the orders of the genetic markers in these regions will be strengthened and the extent of these predicted rearrangements will become better resolved.

**Table 2:** Homologous chromosome arms of Atlantic salmon and rainbow trout**.

**Atlantic Salmon**		**Rainbow Trout**		**Atlantic Salmon**		**Rainbow Trout**	
**Chr**.	**LG**	**Chr**.	**LG**	**Chr**.	**LG**	**Chr**.	**LG**
1p	17	19q	14q	13qa	5	16q	22q
1qa	"	23	30	13qb	"	12p	9p
1qb	"	5p	8p	14qa	3	8q	23q
2p	1 (sex)	3p	31p	14qb	"	14p	3p
2q	"	17p	29p	15qa	8	8p	23p
3p	11	28	13	15qb	"	9q	21q
3q	"	12q	9q	16qa	23	1p	6p
4p	28	19p	14p	16qb	"	18p	16p
4q	"	10p	20p	17qa	22	15q	7q
5p	12	14q	3q	17qb	"	7p	12p
5q	"	2p	27p	18qa	16	1q	6q
6p	4	4q	24q	18qb	"	9p	21p
6q	"	13q	2q	19qa	13	11p	19p
7p	24	21p	15p	19qb	"	16p	22p
7q	"	21q	15q	20qa	25	11q	19q
8p*	rDNA	20p*	rDNA	20qb	"	27	11
8q	19	10q	20q	21	14	22p, q	5
9qa	10	25	4	22	32	7q	12q
9qb	"	29	25	23	18	4p	24p
9qc	"	24	26	24	7	6p	10p
10qa	2	5q	8q	25	20	3q	31q
10qb	"	2q	27q	26	21	6q	10q
11qa	9	sex	1	27	15	18q	16q
11qb	"	26	18	28	33	20q	17q
12qa	6	13p/17p	2p/29p	29	31	15p	7p
12qb	"	17q	29q				

*Note: The short arm of chromosome 8 (LG 19p) in Atlantic salmon and chromosome 20 (LG 17p) in rainbow trout are composed entirely of ribosomal DNA, and so are not counted as one of the 50 haploid chromosome arms. Table 2 is an updated version of part of Table 1 [11] with a couple of corrections. In that table, the homologies between rainbow trout 7p and 7q were assigned an inverted orientation. As shown here, rainbow trout LG 7p corresponds to Atlantic salmon LG 31 whereas rainbow trout LG 7q corresponds to Atlantic salmon LG 22qa. In addition, Atlantic salmon LG 1q corresponds to rainbow trout LG 29p (i.e., RT-29 arms were previously reported in an inverted orientation).

** It should be noted that there are only 29 linkage groups in the Atlantic salmon genetic map. The nomenclature for the linkage groups is historical, and linkage groups 26, 27, 29 and 30 no longer exist. Similarly, there are only 30 linkage groups in rainbow trout as linkage group 28 no longer exists.

## Discussion and conclusion

Atlantic salmon has a karyotype with either 74 chromosome arms in the European subspecies or 72 chromosome arms in the North American subspecies compared to approximately 100 chromosome arms found in the karyotypes of other salmonid species in the subfamily Salmoninae [4]. Although rainbow trout has traditionally been assigned an NF of 104, if we ignore the short arm of chromosome 20 which is composed entirely of ribosomal DNA and consider the smallest metacentric chromosome as a single arm which underwent a pericentric inversion (i.e., Chr 22p, q), then rainbow trout has 100 chromosome arms as well, as do all the other *Oncorhynchus *species. When the rainbow trout chromosomes are arranged according to size, the smallest metacentric chromosome pair (LG 5) falls between the largest and second largest acrocentrics, a result which supports the inversion hypothesis for this chromosome. In addition, rainbow trout LG 5 corresponds to a single chromosome arm in Atlantic salmon and in the ancestral vertebrates [11]. As described above, the Atlantic salmon karyotype has 12 large acrocentric chromosomes that appear to have undergone tandem fusions [4]. The largest acrocentric chromosome (Chr. 9, LG 10) has two interstitial bands, dividing the chromosome into three sections and suggesting it may be the result of two such tandem fusions. Moreover, the largest metacentric chromosome (Chr. 1, LG 17) has an interstitial band in the long arm, suggesting it too may have undergone a tandem fusion. Thus, 14 tandem fusions would involve 28 original chromosome arms. When these 28 chromosome arms are added to the 74 observed in the European Atlantic salmon karyotype, we get 102 arms. However, the smallest metacentric chromosome has a short arm composed entirely of ribosomal DNA [7], so when this arm is subtracted, we obtain 100 chromosome arms, the same number as rainbow trout and the other *Oncorhynchus *species. When the location of genetic loci mapped in rainbow trout are indicated on the Atlantic salmon merged female genetic map of the Br5 and Br6 SALMAP families (Additional File 2) and the two male maps (Additional File 3 and Additional File 4), it can be seen that there is an excellent correspondence between the 50 diploid chromosome arms or equivalent segments in each species, including the order of the markers in most cases (Additional File 5). Therefore, we conclude that the karyotypes of both species are composed of the same 50 large syntenic blocks, which correspond to extant or previous chromosome arms in the haploid karyotypes. The locations of these syntenic blocks are shown in Table 2.

The salmonids underwent adaptive radiation following the whole genome duplication of their common ancestor, which probably had a typical teleost karyotype comprising 25 pairs of acrocentric chromosomes [5], and so we would expect that 25 pairs of duplicated segments might be identified in each species. Although 27 duplicated marker regions have been identified among different rainbow trout linkage groups, and a similar number (26) have been identified in Atlantic salmon, only 8 of these duplicated sets in Atlantic salmon involve two or more genetic markers. It has been hypothesized that this may be related to the great reduction in genetic recombination in Atlantic salmon males, which in turn could be related to the tandem fusions. Pairing of homeologous chromosomes involving tandem fusions would lead to abnormal gametes following crossing-over, compared to the normal gametes produced by crossing-over in paired homeologous chromosomes that involved centric fusions. The absence of recombinant gametes could lead to accumulation of mutations, silencing of one of the duplicate loci or divergence of duplicated loci, and so they might be harder to identify. One possible test of this hypothesis is to examine the location of the currently identified homeologous chromosome arms in Atlantic salmon. Table 3 shows the locations of the homeologies with the highest support in rainbow trout and Atlantic salmon. There are seven homeologies in rainbow trout that are supported by nine or more pairs of duplicated markers, and six homeologies in Atlantic salmon that are supported by four or more pairs of duplicated markers. Most of these homeologies involve pairs of metacentric chromosomes. The largest numbers of confirmed homeologies in Atlantic salmon are between LG 1p and LG 12q (both metacentrics) and LG 4p and LG 11p (both metacentrics) [11]. Thus, the majority of these duplicated affinities involve metacentric chromosome pairs. Although the exact mechanism whereby duplications should be preferentially retained within metacentrics is currently unknown, it is evident from the current study that the overall genetic map size of metacentric chromosomes appears to be somewhat elevated compared to acrocentric based pairs. There is one homeologous pair in rainbow trout (LG 10q and LG 18), which is supported by ten duplicated markers, that does not appear to have a counterpart in Atlantic salmon. The homologous chromosome arms in Atlantic salmon would involve a large acrocentric chromosome (LG 9q/Chr 11qb) and a small acrocentric chromosome (LG 21/Chr 26). Therefore, the limited data that are available lend support to the hypothesis that homeologies are preferentially retained between larger metacentric chromosomes compared to small acrocentrics or large acrocentrics that are the result of tandem fusions. Why all of the Atlantic salmon homeologies with strong support are also strongly supported in rainbow trout is unknown, but it may be because diploidization of the other linkage groups occurred in the ancestral salmonid before divergence of the *Salmo *and *Oncorhynchus *lineages. In rainbow trout, about 20% of the BAC clones hybridized to two different chromosomes, usually to loci on the homeologous chromosomes; however, in Atlantic salmon we found only about 3% of BACs hybridized to more than one chromosome pair, which is consistent with the reduction in duplicate markers found in this species.

**Table 3:** Highly supported homeologous chromosome arms in Atlantic salmon and rainbow trout

**AS LG**	**AS Chrom**	**RT LG**	**RT Chrom**.	**#duplicated markers**	
				**AS**	**RT**
1q/6q	2q/12qb	2p/29p	13p/17p	5	13
1p/12q	2p/5q	31p/27p	3p/2p	9	16
4p/11p	6p/3q	9q/2q	12q/13q	9	20
22q/24q	17qa/7q	7q/15q	15q/21q	5	9
22q/23	17qb/16qa	12p/16p	7p/18p	5	15
19q/28p	8q/4p	20q/14p	10q/19p	4	10
21/9q*	26/11qb	10q/18	6q/26	0	10

Note: All pairs with duplicated markers in rainbow trout except the last pair involve two metacentric chromosomes. *This last pair does not contain duplicated markers in Atlantic salmon and involves a small acrocentric and large acrocentric in that species. Although LG 18 is an acrocentric chromosome in rainbow trout, it is part of a metacentric in coho and chinook salmon, suggesting that this may be the ancestral configuration for the genus *Oncorhynchus*.

Affinities to the putative ancestral vertebrate chromosome arms with homologous segments within the Atlantic salmon and rainbow trout genomes have recently been described [11]. These chromosome arm assignments are based upon the model of Nakatani et al. [16], which outlines the evolution of two rounds of whole genome duplicated proto-vertebrate chromosomes that underwent extensive fusions prior to a third round of whole genome duplications before the radiation of the teleost fishes. According to this model, the 10–13 proto-vertebrate linkage groups following two rounds of whole genome duplication may have generated 40–52 distinct chromosomal segments. These segments were then postulated to have undergone extensive fusions to give rise to a 13 chromosome (designated A-M) proto-Actinopterygian linkage group set, which in turn would generate two linkage groups belonging to each ancestral group following the 3R teleost genome duplication. In salmonids, which underwent an additional whole genome polyploidization event [1], evidence has been provided that each of the ancestral proto-Actinopterygian linkage group arms may be represented by up to 4 whole-arm or more partial arm affinities in rainbow trout and Atlantic salmon [11], which would be expected in a 4R-derivative genome. Support was also provided that the M linkage grouping was derived from a triplication event [17], as 5 whole-arm plus 3 partial arm affinities were detected in the salmonids for this grouping. It has also been reported that the putative GH grouping may have undergone more extensive mixing of segments with the I ancestral grouping, as arm assignments within these clusters were not as clearly resolved within the salmonids as was evident for other ancestral groupings [11]. It was suggested that these re-arrangements may have occurred subsequent to the 3R duplication and hence may be of younger evolutionary origin.

In the context of this model it is interesting to note that 8 of the 9 larger acrocentric chromosome arms in Atlantic salmon, which appear to have arisen through the fusion of two or more whole acrocentric chromosomes, involve at least one chromosome arm from the GH/I or M ancestral groupings. For example, LG 10/Chr 9 appears to be a fusion among GH/A segments, while LG 2/Chr 10, LG 9/Chr 11, LG 5/Chr 13, LG 3/Chr 14, LG 23/Chr 16, LG 13/Chr19, and LG 25/Chr20 involve segments from: M/J/K; GH/I/J; GH/I/D/L; B/M; M/C/J/K; D/E/M and GH/I, respectively. It should be noted that for LG 10/Chr2, which appears to involve three whole-arm tandem fusions, two of these arms are derived from the GH grouping. The only acrocentric undergoing a fusion that appears not to possess any element from the GH/I/M segments is LG 6/Chr 12. This linkage group appears to only possess D/L derived segments. Segments from two other fused arm acrocentric chromosomes (i.e., LGs 16 and 22) currently have unknown homologies to complete arm segments [11] and thus cannot be categorized. Examination of genetic maps from other salmonids shows that segments derived from the GH, I and M groupings along with A/F/L lineages are not involved in multivalent formations during meiosis I (i.e., pseudolinkage). Such formations are more typical among linkage groups belonging to the B/C/D/E/J/K ancestral lineages and these differences may also relate to the distribution of transposable elements throughout the chromosome arms of salmonids [11]. Linkage groups expressing pseudolinkage retain a higher distribution of duplicated marker copies and also appear to possess a lower density of transposable element fragments. Thus, the lack of a propensity to form multivalent structures accompanied by the loss of duplicate genes may be a prerequisite to stabilize initial acrocentric fusions if they arise in the evolution of a lineage.

## Methods

### Atlantic salmon BACs containing BAC-end microsatellites

A total of 207,869 BAC-end sequences with an average length of 666 bp were screened for microsatellites using a Perl script created in the Davidson Lab, and 20,606 microsatellites were identified. As of December 2008, 697 of these microsatellite markers have been placed on the Atlantic salmon genetic map using the SALMAP mapping families Br5 and Br6 [18,19] and the LINKMFEX package [20].

### BAC library screening for microsatellites on the linkage map

Oligonucleotide probes (35–40 mer), which also acted as forward primers for PCR, and 20 mer reverse complement PCR primers were designed using primer3 software  from the flanking regions surrounding microsatellite markers identified as anchor loci on the Atlantic salmon genetic map. The resulting sequences were subsequently screened for suspected and known repetitive elements in salmonids using a salmonid-specific repeat masker . An additional probe (5'-GTTGCCAAATTCCGAGATCTTGGCGACGAAGCCACATGAT-3') was used to hybridize to the *C. briggsae *control anchor spots on the CHORI-214 BAC filters. Oligonucleotide probes were radioactively labeled at the 5' end with ^32^Pγ-ATP and T4 polynucleotide kinase (Invitrogen) in a 10 μL volume reaction at 37°C for 30 minutes. The labeling reaction included 1 μL of 10 μM probe, 10 U of T4 polynucleotide kinase, 2 μL of 5× forward reaction buffer, 2 μL of ^32^Pγ-ATP (3000 Ci/mmol) and 4 μL of water. Each of the CHORI-214 high density BAC filters was pre-hybridized for 3 hours in 50 or 100 ml of pre-hybridization solution (5× SSC, 0.5% SDS, 5× Denhardt's solution) at 65°C. To each pre-hybridized filter 1.6 μL of radioactively labeled oligonucleotide probe and 1.6 μL of radioactively labeled *C. briggsae *overgo reference probe were added and hybridization was carried out at 65°C overnight (~18 hours). Three 1 hour washes consisting of 1× SSC and 0.1% SDS were performed at 50°C to remove unhybridized probe. Filters then were wrapped in Saran™ wrap and exposed on storage phosphor screens for 18 hours. Hybridization signals were detected using a Typhoon 9410 Phosphor Imager (Amersham Biosciences).

### PCR confirmation of BAC clones containing microsatellites

BAC clones, positive by hybridization for a specific microsatellite or identified as containing a BAC-end microsatellite, were identified in the Atlantic salmon physical map . These BAC clones were picked from the CHORI-214 Atlantic salmon BAC library and grown in 5 ml of LB medium containing 20 μg/mL chloramphenicol for 14–16 hours at 37°C with shaking at 250–300 rpm. Glycerol stocks were made by placing 700 μL of each culture into 1.5 mL Eppendorf™ tubes containing 300 μL of 50% glycerol. BAC DNA templates for PCR were prepared from selected clones by diluting the glycerol stock 1/40 in dH_2_O. Colony PCR of the selected clones positive by hybridization for a particular microsatellite's flanking region probe was performed in a T3 or T3000 Thermalcycler (Biometra) using appropriate primers and annealing temperature in 25 μL using the following procedure: initial denaturation step of 95°C for 5 min; 35 cycles with a denaturation step of 95°C for 45 sec, 45 sec at specific annealing temperature, extension at 72°C for 45 sec followed by a final extension at 72°C for 10 minutes. The PCR contained 0.05 U Taq DNA Polymerase (Qiagen), 12.5 pmoles of each specific primer, 2.5 μL 10× PCR buffer containing 1.5 mM MgCl_2_, 50 μM dNTP and 5 μL of BAC DNA template. PCR products were separated on a 1.5% agarose gel containing 1× TBE and 0.5 μg/ml ethidium bromide. The DNA fragments were visualized using a UV trans-illuminator (Ultra-Violet Products). Only those BAC clones that yielded clean single amplification products of the expected size were used for FISH analysis.

### *In situ *hybridization and karyotyping

Chromosome preparations were obtained from blood of the European strain of Atlantic salmon (2N = 58) by methods described previously [21]. Briefly, the buffy coat was isolated from whole blood and placed in MEM media with pen-strep, L-glutamine, 10% fetal calf serum and 200 ug/ml LPS and cultured for 6 days at 20°C. Cells were collected by centrifugation and re-suspended in 0.075 M KCl for 30 minutes, then fixed in 3:1 methanol:acetic acid. Cell suspensions were dropped on to clean slides and allowed to dry on a slide warmer with humidity at 40°C. BAC DNA was isolated from clones containing a microsatellite marker with a known position in the Atlantic salmon linkage map using the QIAGEN Plasmid Midi Kit (Catalog # 12143) following the manufacturer's protocol. BAC DNA was labeled with spectrum orange (Vysis, Inc.) and digoxigenin (Roche, Inc.) as recommended by the manufacturers. Hybridization with fluorochrome-labeled dUTPs was as suggested by the manufacturer (Vysis, Inc.) with minor modifications. Briefly, chromosome preparations were made the day before use and left to dry on a slide warmer at 40°C overnight. Just prior to hybridization, the slides were denatured in a 70% formamide solution at 73°C for 5 minutes. The probe was prepared by adding labeled DNA with human placental DNA and Atlantic salmon or rainbow trout Cot1 DNA (for blocking) to the Vysis hybridization solution, and denatured at 73°C for 5 minutes. Hybridization was allowed to proceed under a sealed coverslip in a humidified chamber at 37°C overnight. The following day the slides were washed first with 0.3% NP40 in 0.4× SSC at 73°C for 3 minutes, and then with 0.1% NP40 in 2× SSC at room temperature for 1 minute. Antibodies to digoxigenin (1/100 dilution in PBS) were applied, and the slides were incubated at 37°C for 45 minutes, according to manufacturer's instructions. Primary and secondary antibodies to spectrum orange (1/100 and 1/200 dilution in PBS) were used to amplify the signal in many experiments. Slides were counterstained with DAPI/antifade (Vysis, Inc.) and then examined using an Olympus BX60 microscope and photographed with either a Sensys or Jai digital camera. Images were captured with Cytovision software (Applied Imaging Inc.) and selected karyotypes were prepared using Genus software (Applied Imaging Inc.). Chromosome pairs were identified using relative size, centromere position and presence of interstitial bands revealed by reverse DAPI staining. Chromosomes were assorted according to size using the software described above and adjustments made by hand to conform with the standard chromosome arm ratios and DAPI staining patterns.

### Comparison of Atlantic salmon and rainbow trout linkage maps and karyotypes

We examined several genetic maps for rainbow trout [18,22-24] for markers found on the consensus female Atlantic salmon map [11]. Two of the rainbow trout maps [23,24] contain 30 linkage groups corresponding to 60 chromosomes, while the other rainbow trout genetic maps [18,22] contain 29 linkage groups corresponding to 58 chromosomes. Interior rainbow trout strains usually have 58 chromosomes, while coastal strains can have from 60 to 64 chromosomes [25]. It has previously been shown that LG 4 and LG 25 from the Nichols et al. map [24], which correspond to acrocentric chromosome pairs 25 (LG 4) and 29 (LG 25) in the OSU rainbow trout strain [22], are fused to form a metacentric chromosome in the strains with 58 chromosomes [26-33]. Therefore, in the genetic linkage maps based on rainbow trout with 58 chromosomes LG 4 is not present as a separate linkage group but rather found as part of LG 25 as expected from the cytogenetic data. For the comparison with Atlantic salmon we used the linkage groups from the strain with 60 chromosomes and 30 linkage groups. However, all rainbow trout genetic maps were searched for genetic markers found on the consensus female Atlantic salmon map, and the trout linkage group for each of these shared markers is shown on the linkage map of each Atlantic salmon chromosome in Additional File 5.

## Authors' contributions

KPL and RGD constructed the linkage map and integrated the markers with the BAC-based physical map. KAK, MRM, ABV and RBP carried out the FISH and chromosomal analyses. RGD and RBP correlated the Atlantic salmon and rainbow trout linkage maps and homologous chromosome arms. WSD, RBP, RGD and BFP conceived of the study. WSD, RBP and RGD drafted and prepared the final manuscript. All authors read and approved the final manuscript.

## Supplementary Material

Additional file 1 **Table **1: **Assignment of Atlantic salmon genetic linkage groups to chromosomes**. This table shows the relationships of the genetic markers from each of the linkage groups, the BACs that contain the specific markers and the chromosome arm that each of the BACs was assigned to using FISH analysis. Click here for file

Additional file 2 **Figure **1. **Atlantic salmon consensus female genetic map from Br5 and Br6 families**. This figure shows the female genetic map that was constructed based on the SALMAP Atlantic salmon mapping families, Br5 and Br6. Click here for file

Additional file 3 **Figure **2. **Atlantic salmon male genetic map from the Br5 family**. This figure shows the male genetic map that was constructed based on the SALMAP Atlantic salmon Br5 mapping family. Click here for file

Additional file 4 **Figure **3. **Atlantic salmon male genetic map from the Br6 family**. This figure shows the male genetic map that was constructed based on the SALMAP Atlantic salmon Br6 mapping family. Click here for file

Additional file 5 **Table **2. **Location of shared markers on Atlantic salmon and rainbow trout chromosome arms**. This table shows the locations of the genetic markers that are shared in the Atlantic salmon and rainbow trout linkage maps and indicates their chromosomal positions in these species. Click here for file
