# Supplementary material for: Assignment of Atlantic salmon (Salmo salar) linkage groups to specific chromosomes: Conservation of large syntenic blocks corresponding to whole chromosome arms in rainbow trout (Oncorhynchus mykiss)
Source: BMC Genet. 2009 Aug 18;10:46. doi: 10.1186/1471-2156-10-46 (PMC2734554; doi:10.1186/1471-2156-10-46)
Supplement: Additional file 1 — Table 1: Assignment of Atlantic salmon genetic linkage groups to chromosomes. This table shows the relationships of the genetic markers from each of the linkage groups, the BACs that contain the specific markers and the chromosome arm that each of the BACs was assigned to using FISH analysis. [file 1471-2156-10-46-S1.doc]

## Additional file 1: Table 1: Assignment of Atlantic salmon genetic linkage groups* to chromosomes.

**LG Chrom Arm Marker BAC Clone Gene Reference**

1 2 (sex) 2p OmyFgt8TUF S0227A12 14

2p Oneu18ASC S0119E21 14

2p Oneu102ADFG S0018G23 14

2q BHMS150 S0336N24 14

2q Ssa202DU S0605H01 14

2 10 10qa Ssa0020BSFU S0439A22

10qb Ssa10061BSFU S0069I14 LDH-A2 Lubieniecki et al. (unpublished)

10qb Ssa0003BSFU S0131A06

10qb BHMS201 S0143A06

10qb Ssa0172BSFU^m^ S0188I22 Met-B 27

10qb Ssa0026BSFU S0058E01

10qb Ssa10047BSFU S0116D13 LDH-C Lubieniecki et al. (unpublished)

3 14 14qa Ssa0634BSFU S0249L01 TCRalpha 28

14qb BHMS429 S0205B09

14qb Ssa1027BSFU S0008I14 MHC1B 29

4 6 6p Ssa0043BSFU S0322O19

6q BHMS211 S0003A07

6q Ssa10067BSFU^m^ S0055H05 Globin-1 Quinn et al. (unpublished)

5 13 13qb Ssa0008BSFU S0229A14

13qb OMM1096 S0087D06

6 12 12qb BHMS146 S0195D10

12qb Ssa10046BSFU S0457I14 ora-1,2 30

7 24^a^  24 Ssa0006BSFU S0191E15

8 15 15qa Ssa0051BSFU S0092M14

15qb Ssa197DU S0121A09

9 11 11qa Ssa0982BSFU S0056B24

11qb Ssa0066BSFU S0123C16

10 9 9qa Ssa0420BSFU S0220E22 OR Johnstone et al. (unpublished)

9qb Ssa0038BSFU^m^ S0149K02

9qc SSOSL85 S0040B06

9qc Ssa10050BSFU S0136C02 OlfC 31

11 3 3p Ssa0017BSFU S0393B23

3q Ssa86 S0034P12

3q Ssa0516BSFU S0155C07 Globin-2 Quinn et al. (unpublished)

12 5 5p BHMS206 S0026A22

5q BHMS291 S0236C16

13 19 19qb Ssa0609BSFU S0046N23

19qb BHMS289 S0006P09 OR Johnstone et al. (unpublished)

14 21^a^ 21q Ssa0402BSFU S0250B12 Myostatin-2 Lubieniecki et al. (unpublished)

21q Ssa0190BSFU S0033H18

21q BHMS311 S0245I09

15 27 ^a^ 27q Ssa1033BSFU S0030C23 MHC1A 29

27q BHMS127 S0166I14

16 18 18qa Ssa0125BSFU S0036I05

18qa Ssa0192BSFU S0010I02

17 1 1p BHMS281 S0198E23

1qb Ssa0002BSFU S0088O23

1qb Ssa0033BSFU S0274J17 Lysozyme Lubieniecki et al. (unpublished)

18 23^a^ 23q BHMS377 S0102N22

23q Ssa85DU S0286L15

19 8 8p rDNA

8q Ssa10060BSFU S0065J23 FABP2a1 Lai et al. (unpublished)

8q BHMS205 S0351G08

8q Ssa0007BSFU^m^ S0214J02

20 25^a^ 25q Ssa0283BSFU S0179L09 Myostatin-1 Lubieniecki et al. (unpublished)

25q Ssa0187BSFU S0105M10

25q BHMS241 S0014L21

21 26^a^ 26q Ssa0800BSFU S0059P02

26q Ssa0735BSFU S0151P10

22 17 17qa Ssa68NUIG^m^ S0148O07

17qb Ssa0034BSFU S0276I15 LDH-B2 Lubieniecki et al. (unpublished)

17qb BHMS304 S0031H11

23 16 16qa SsaF43NUIG S0216J09 32

16qa Ssa10063BSFU S0052D03 LDH-A1 Lubieniecki et al. (unpublished)

16qa Ssa1020BSFU S0085O16 Met-A 27

16qb OMM1013 S0097E23

24 7 7p Ssa0032BSFU S0225J21 LDH-B1 Lubieniecki et al. (unpublished)

7p Ssa0023BSFU S0518G09

7q BHMS179^m^ S0222K14

25 20 20qa Ssa0730BSFU S0067N15

20qb Ssa9 S0118K06

28 4 4p Ssa0133BSFU/i S0426K01

4q Ssa405UoS S0017A21

31 29^a^ 29q Ssa0084BSFU S0016D16

32 22^a^ 22q Ssa0159BSFU S0332F21 MHCII 33

22q Ssa65 S0018L24

22q Ssa0617BSFU S0487M08 OR Johnstone et al. (unpublished)

33 28^a^ 28q Ssa224 S0021G10

28q Ssa0001BSFU^m^ S0091M08

^*^ The nomenclature for the linkage groups is historical, and linkage groups 26, 27, 29 and 30 no longer exist (i.e., they were recognized as parts of larger linkage groups as more mapping data were added). ^a^ small acrocentric chromosome ^m^ Found on male map only OR = Odorant Receptor
