# Supplementary figures and images for: Assignment of Atlantic salmon (Salmo salar) linkage groups to specific chromosomes: Conservation of large syntenic blocks corresponding to whole chromosome arms in rainbow trout (Oncorhynchus mykiss)
Source: BMC Genet. 2009 Aug 18;10:46. doi: 10.1186/1471-2156-10-46 (PMC2734554; doi:10.1186/1471-2156-10-46)

## Slide 1
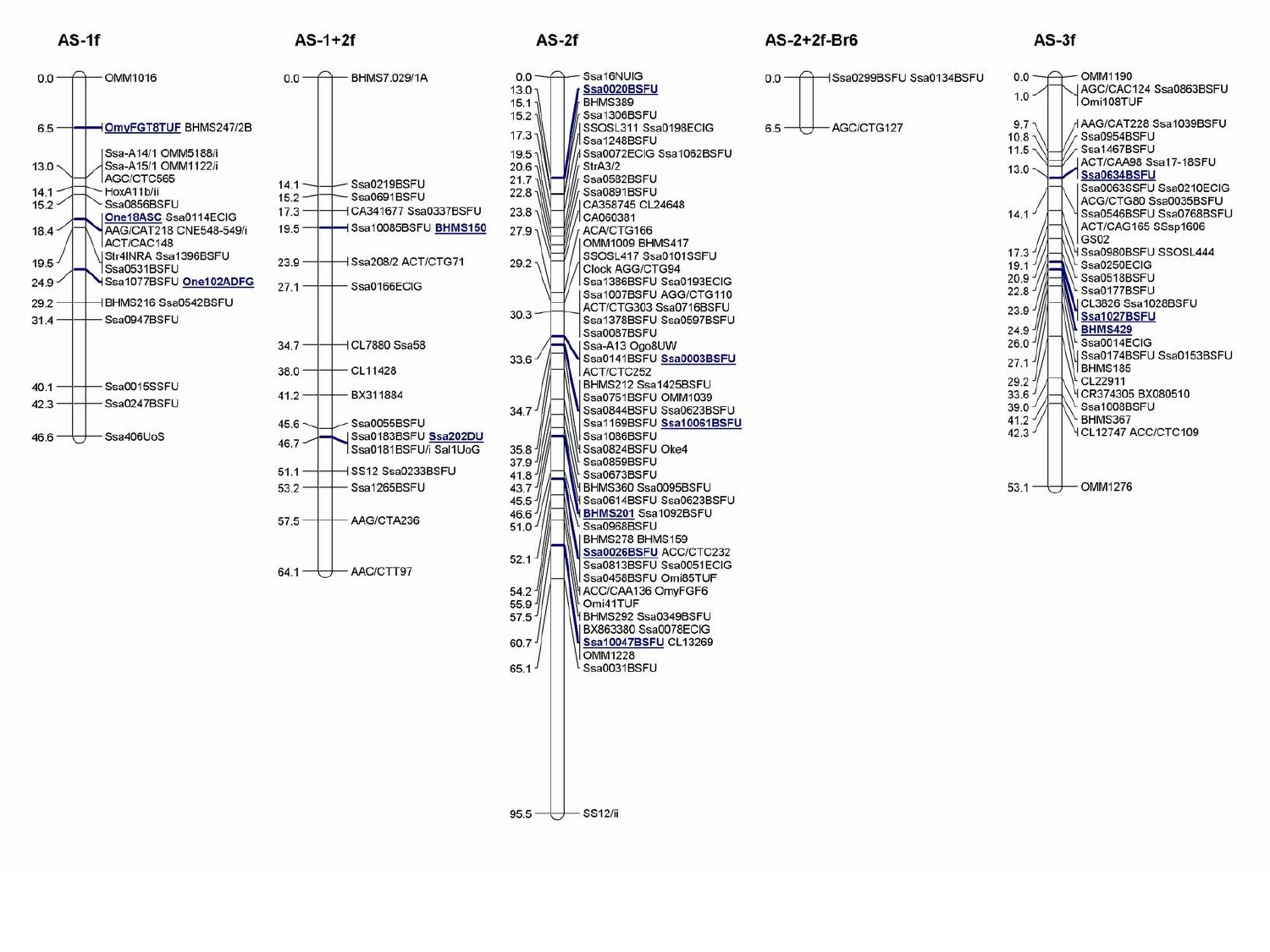

## Slide 2
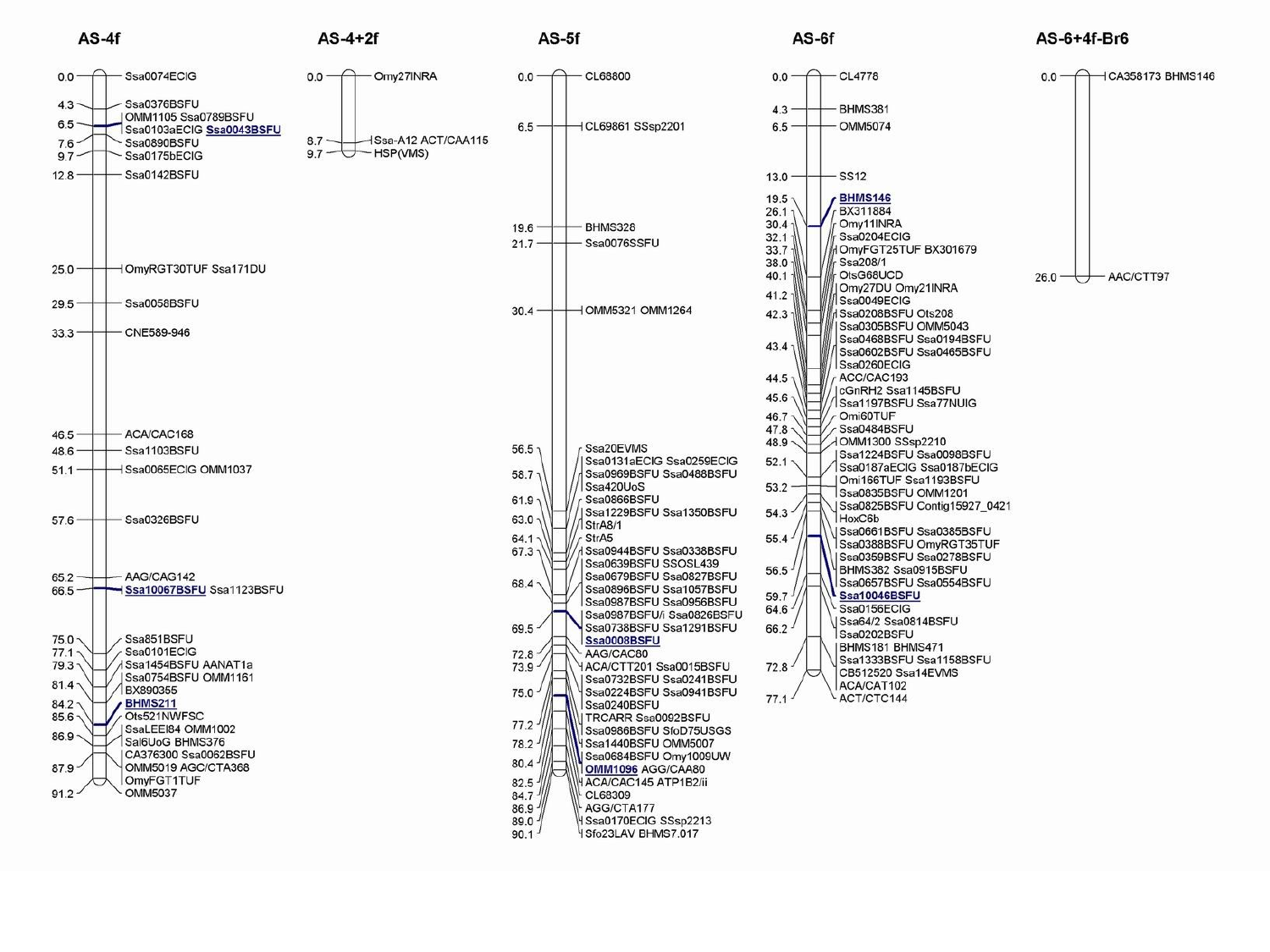

## Slide 3
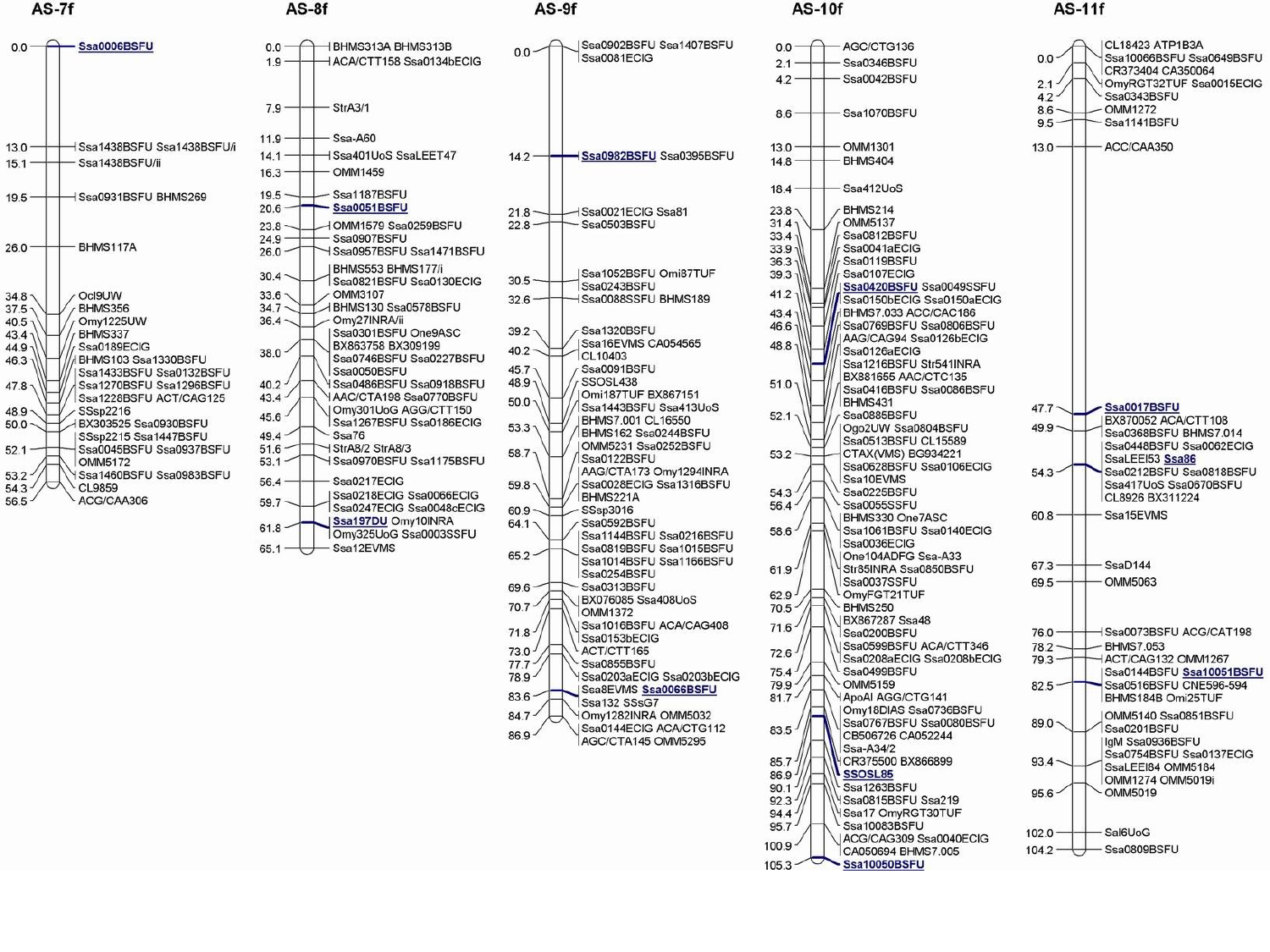

## Slide 4
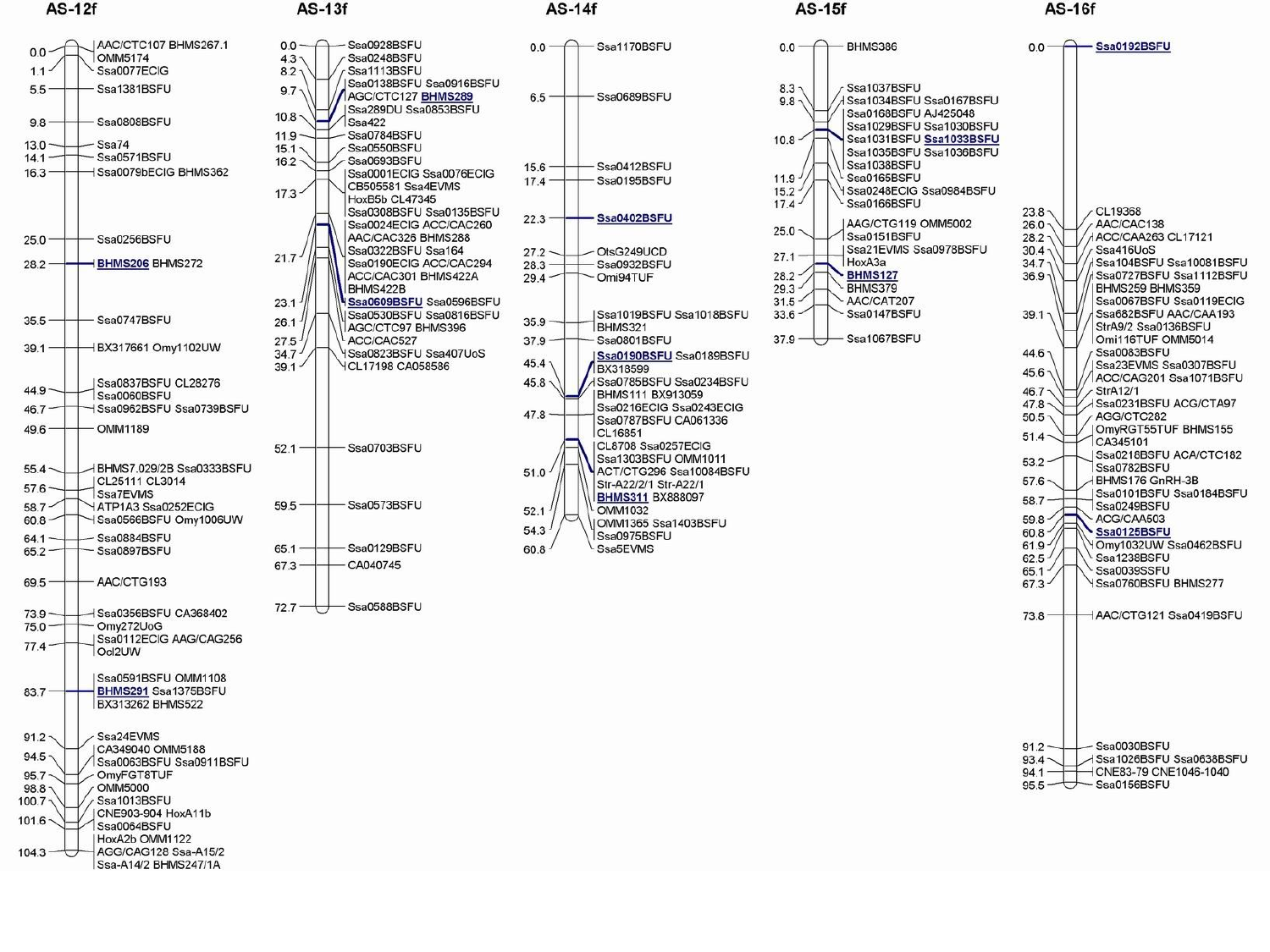

## Slide 5
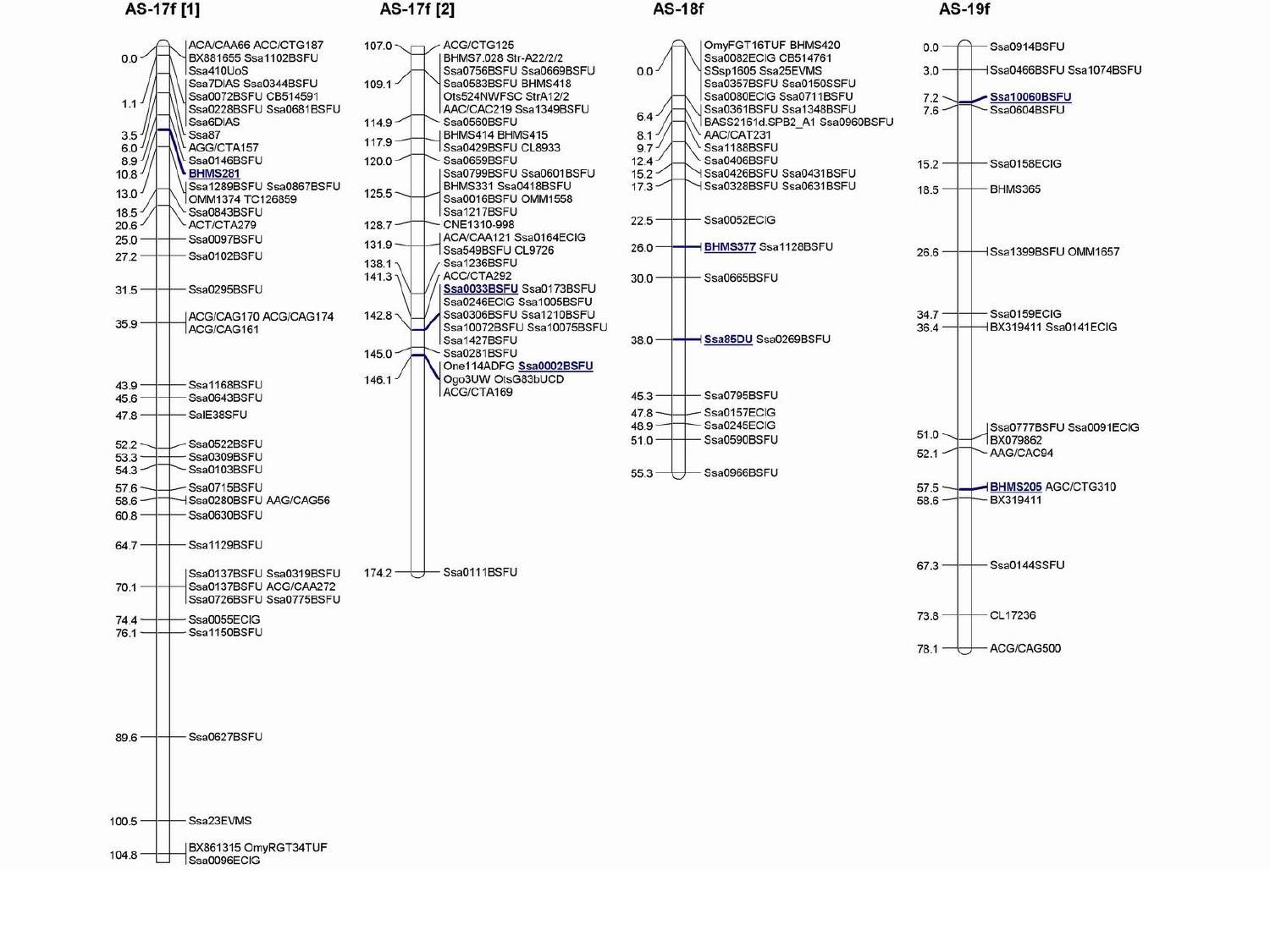

## Slide 6
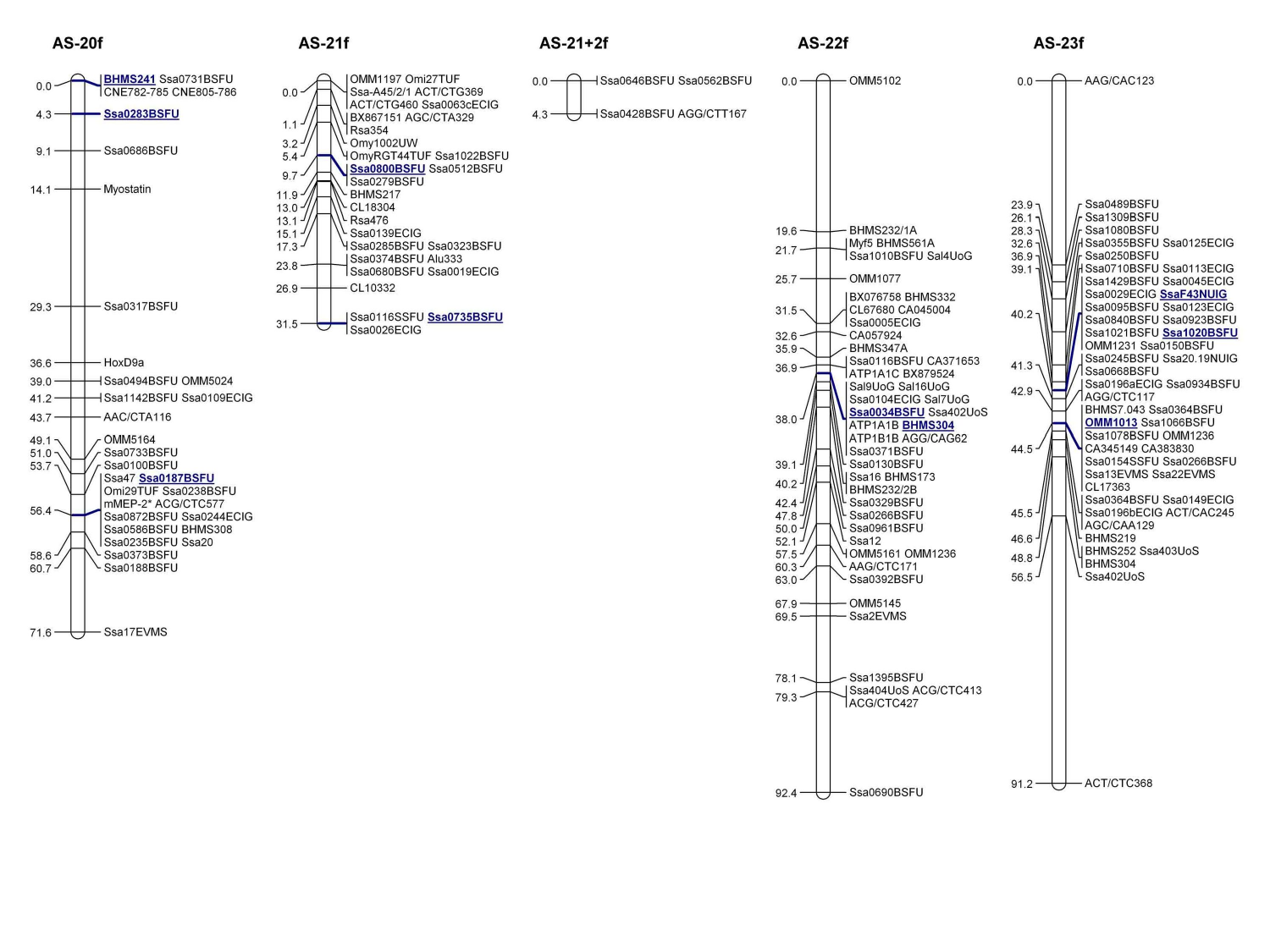

## Slide 7
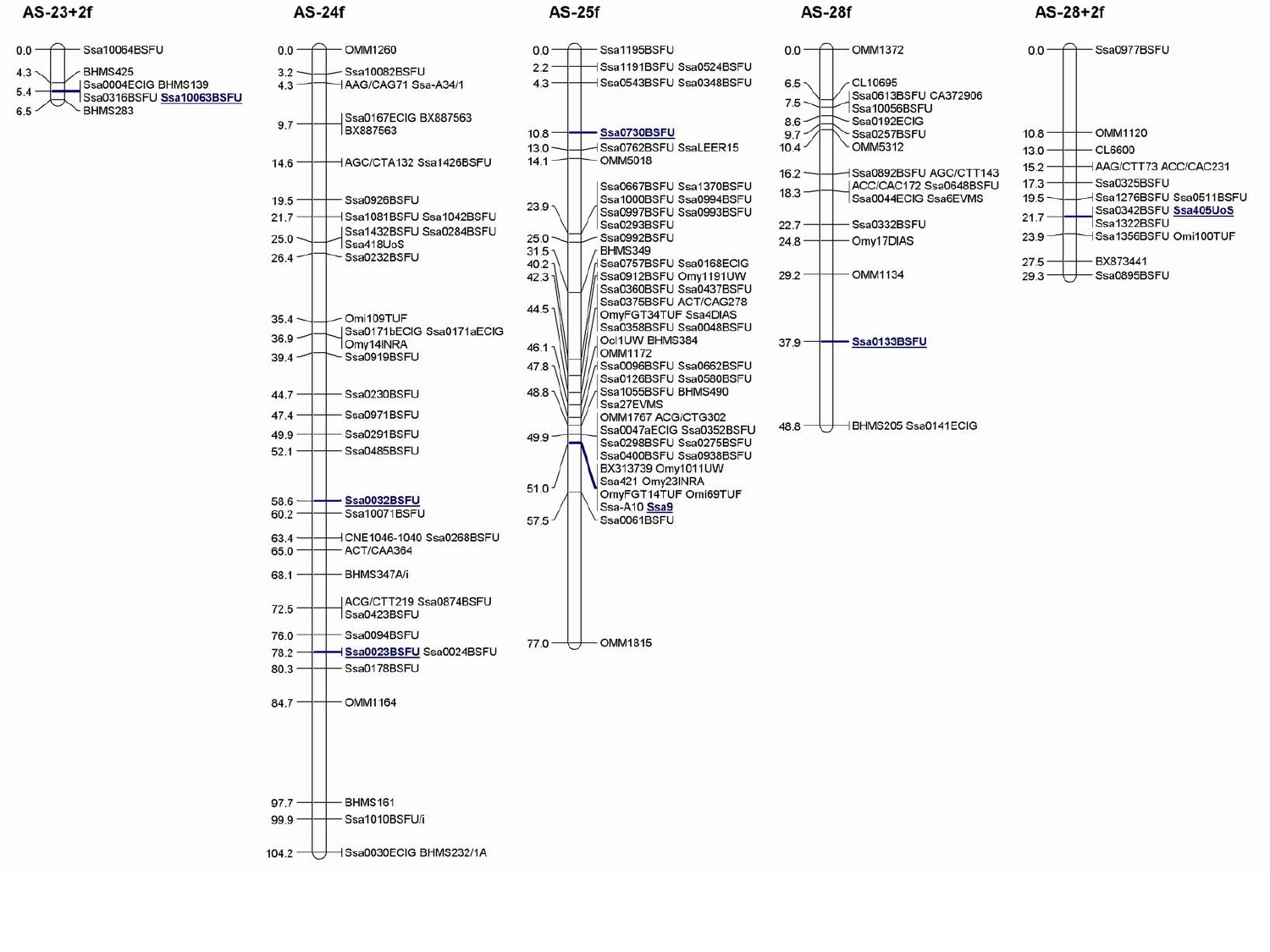

## Slide 8
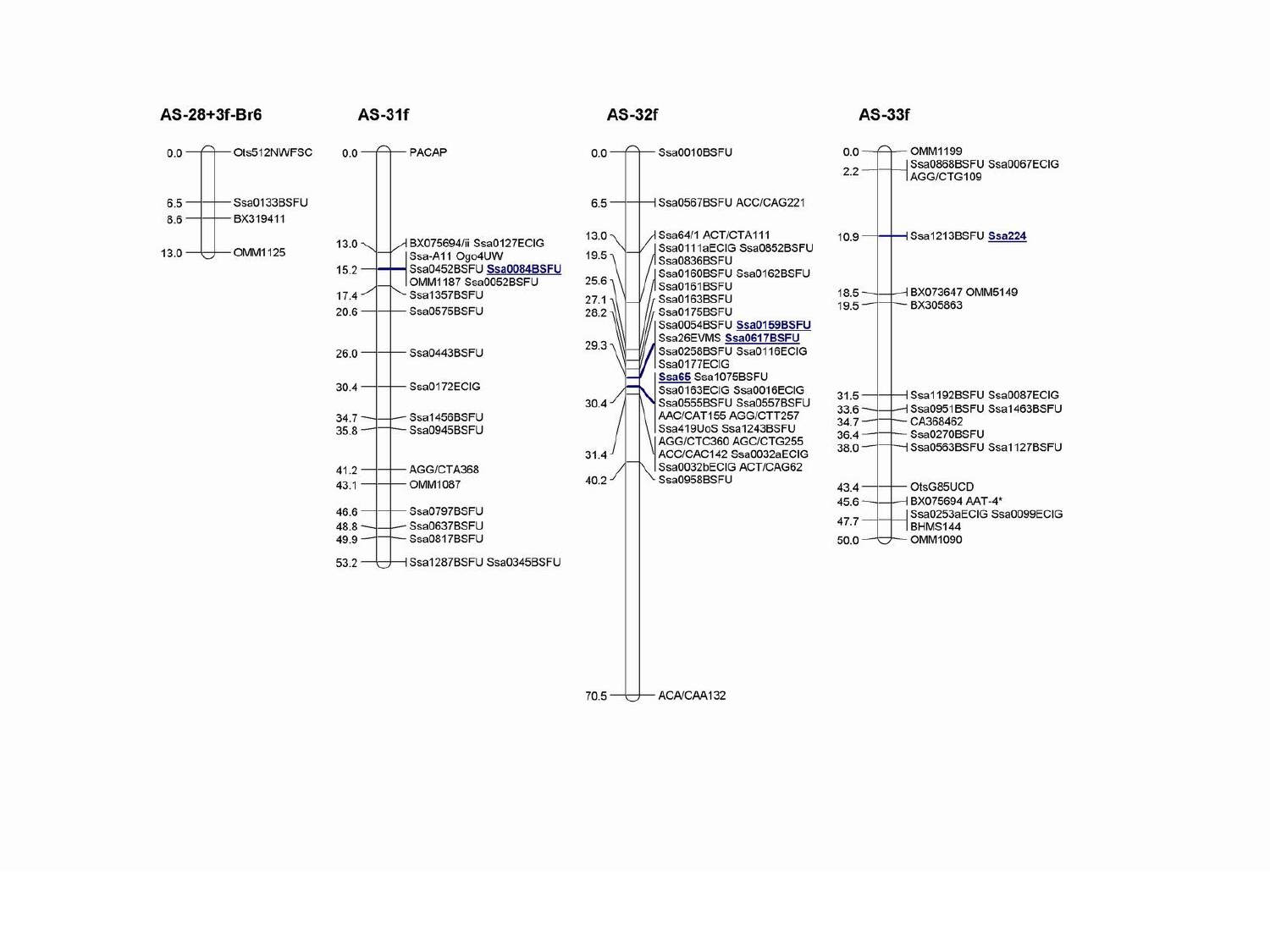

Supplement: Additional file 2 — Figure 1. Atlantic salmon consensus female genetic map from Br5 and Br6 families. This figure shows the female genetic map that was constructed based on the SALMAP Atlantic salmon mapping families, Br5 and Br6. [file 1471-2156-10-46-S2.ppt]

## Slide 1
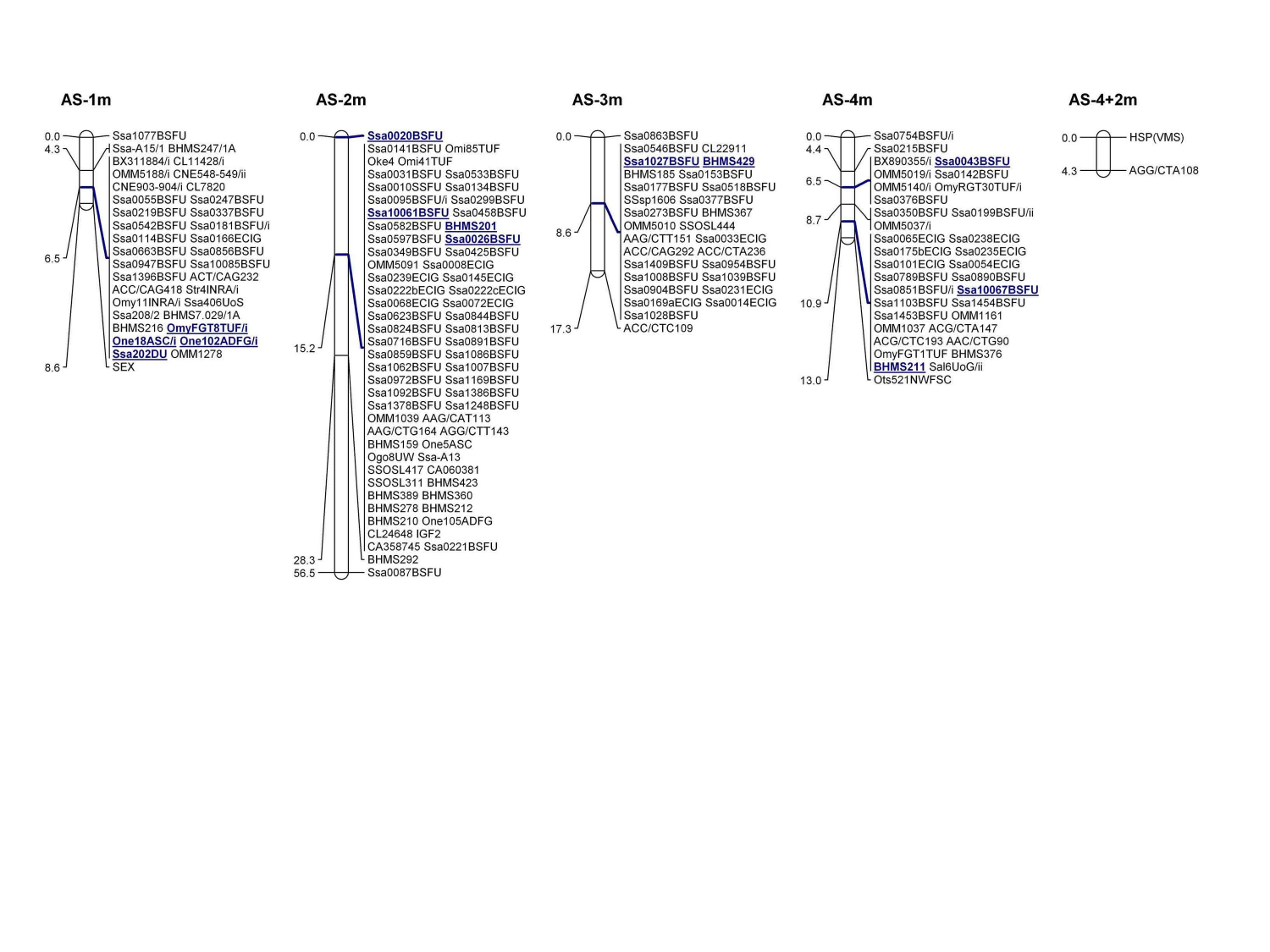

## Slide 2
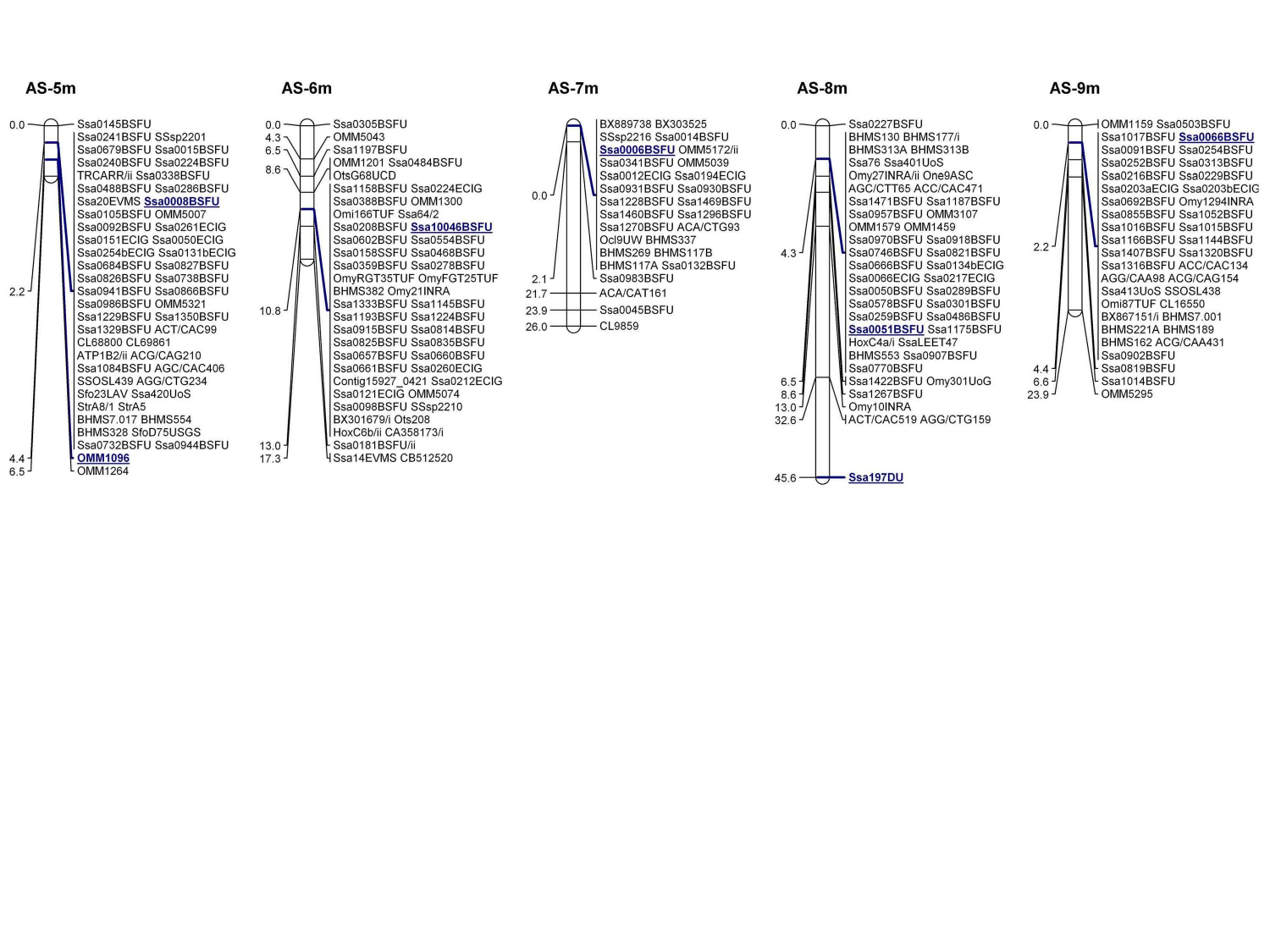

## Slide 3
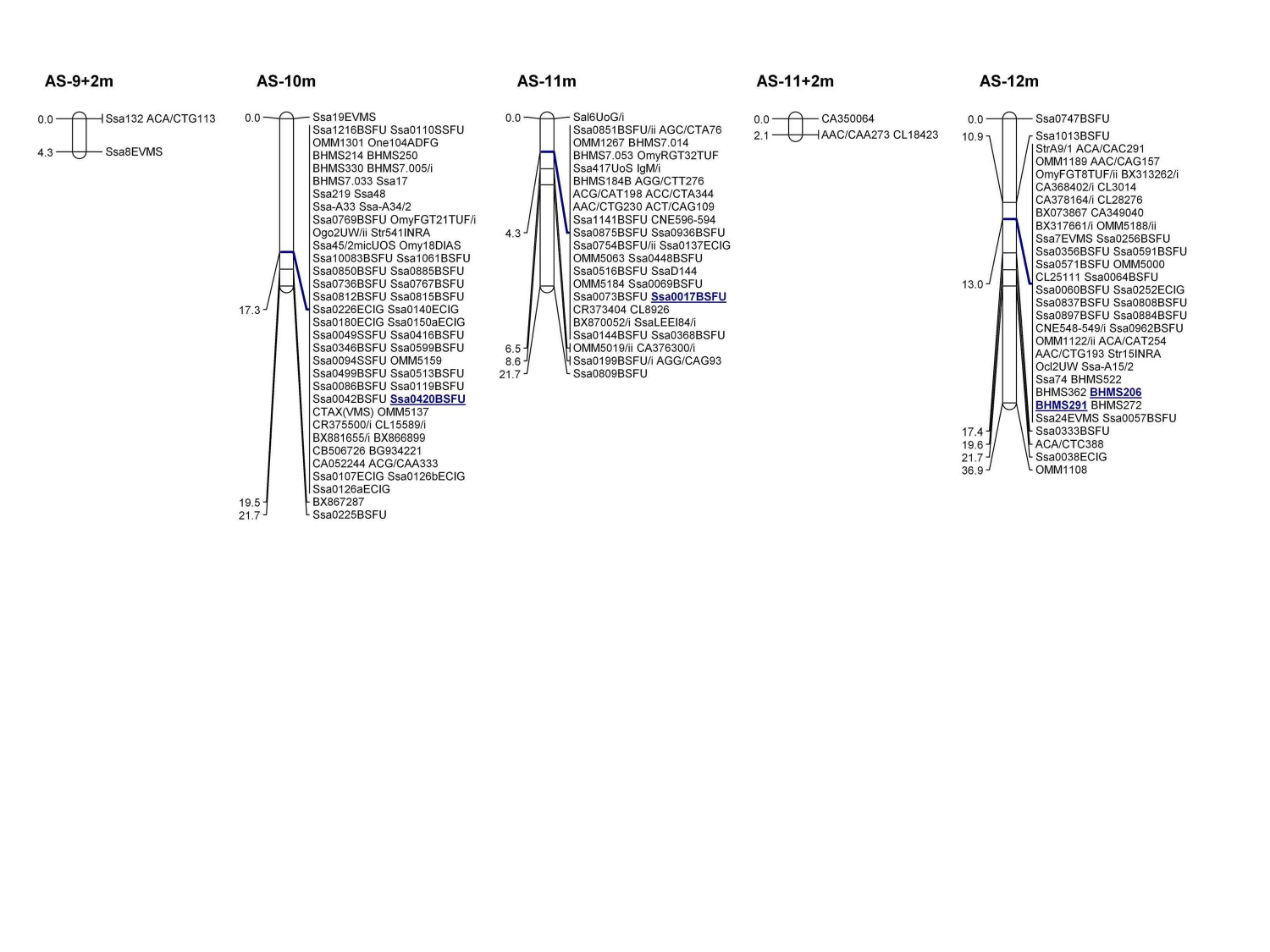

## Slide 4
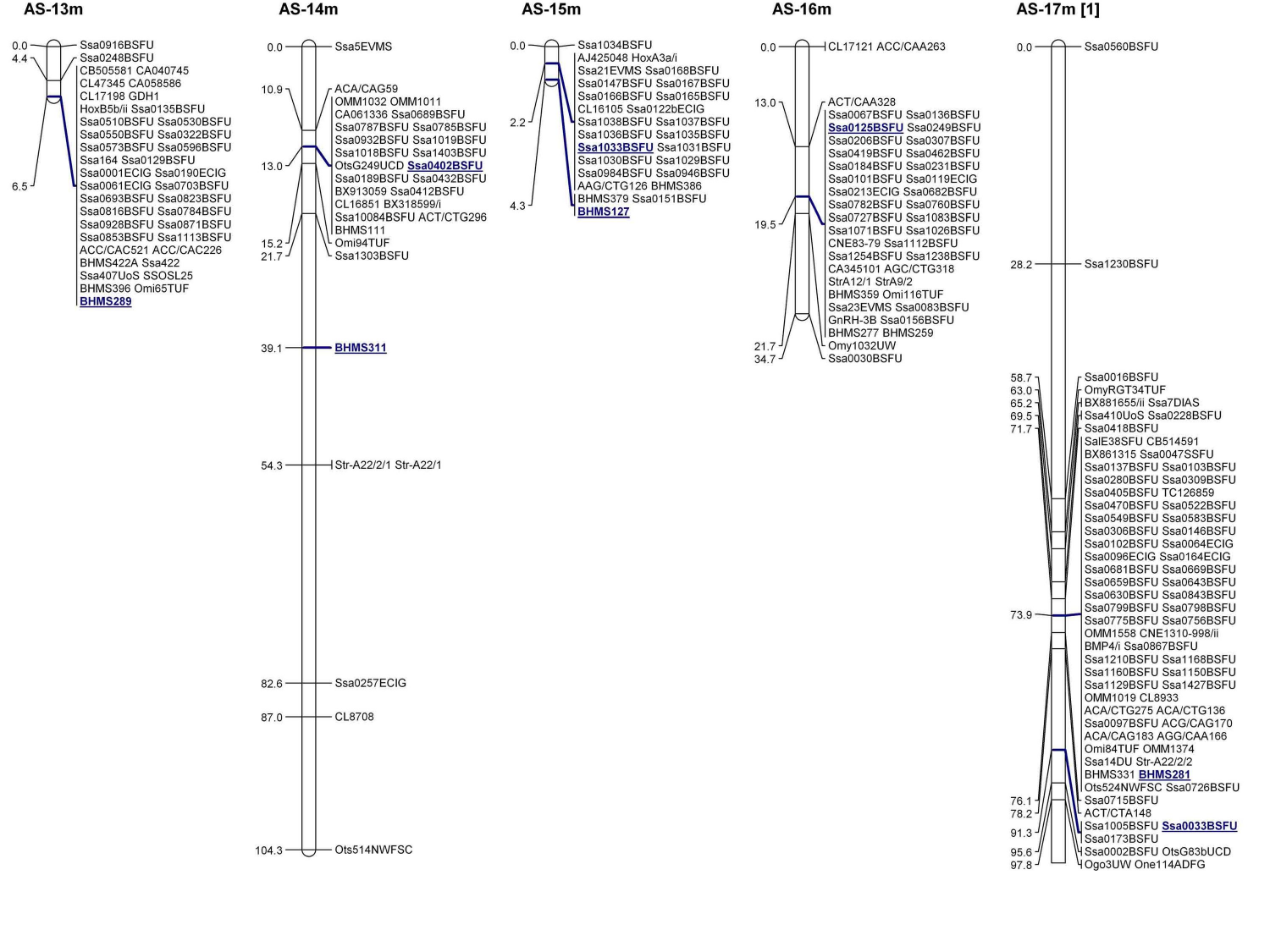

## Slide 5
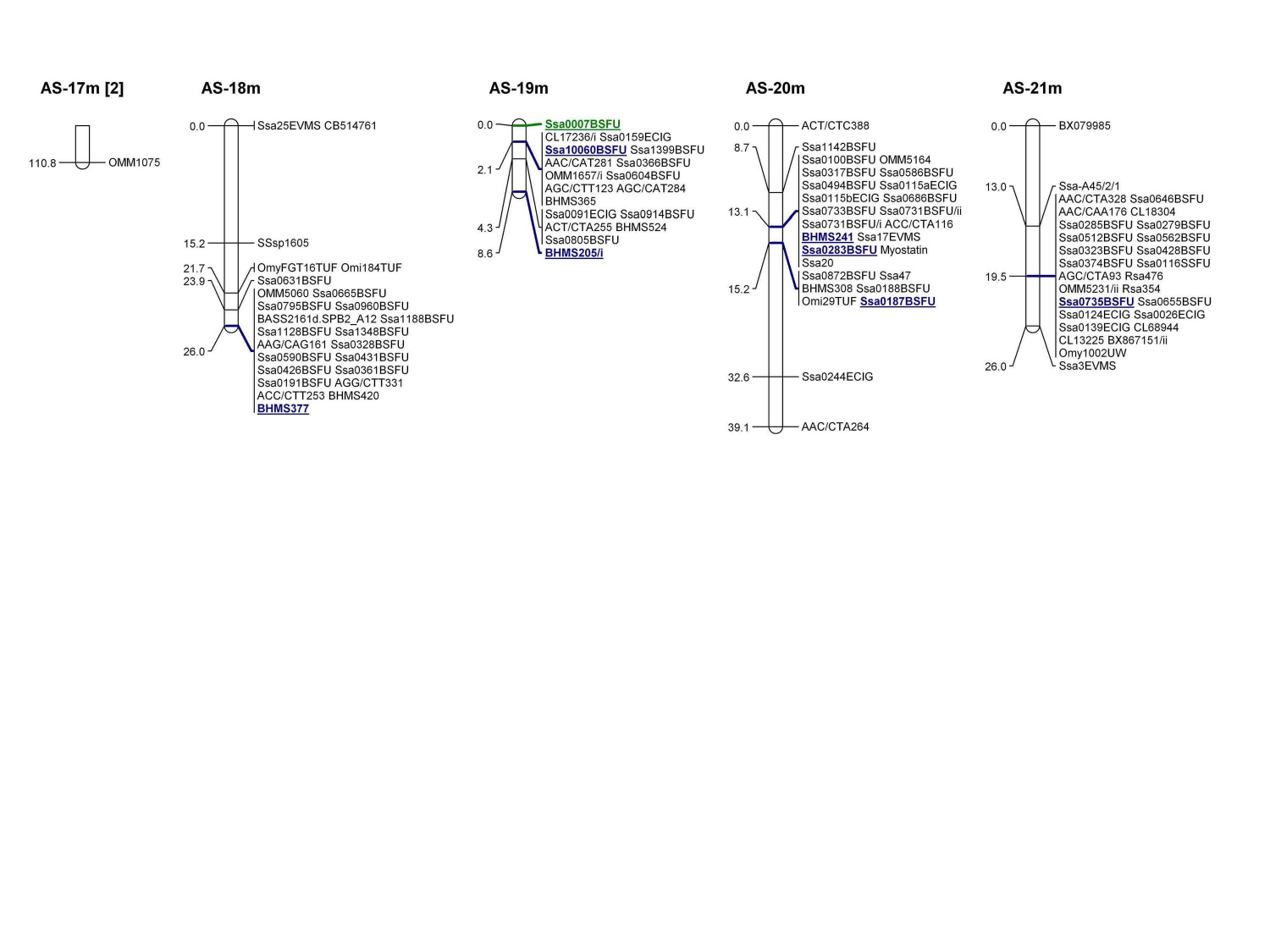

## Slide 6
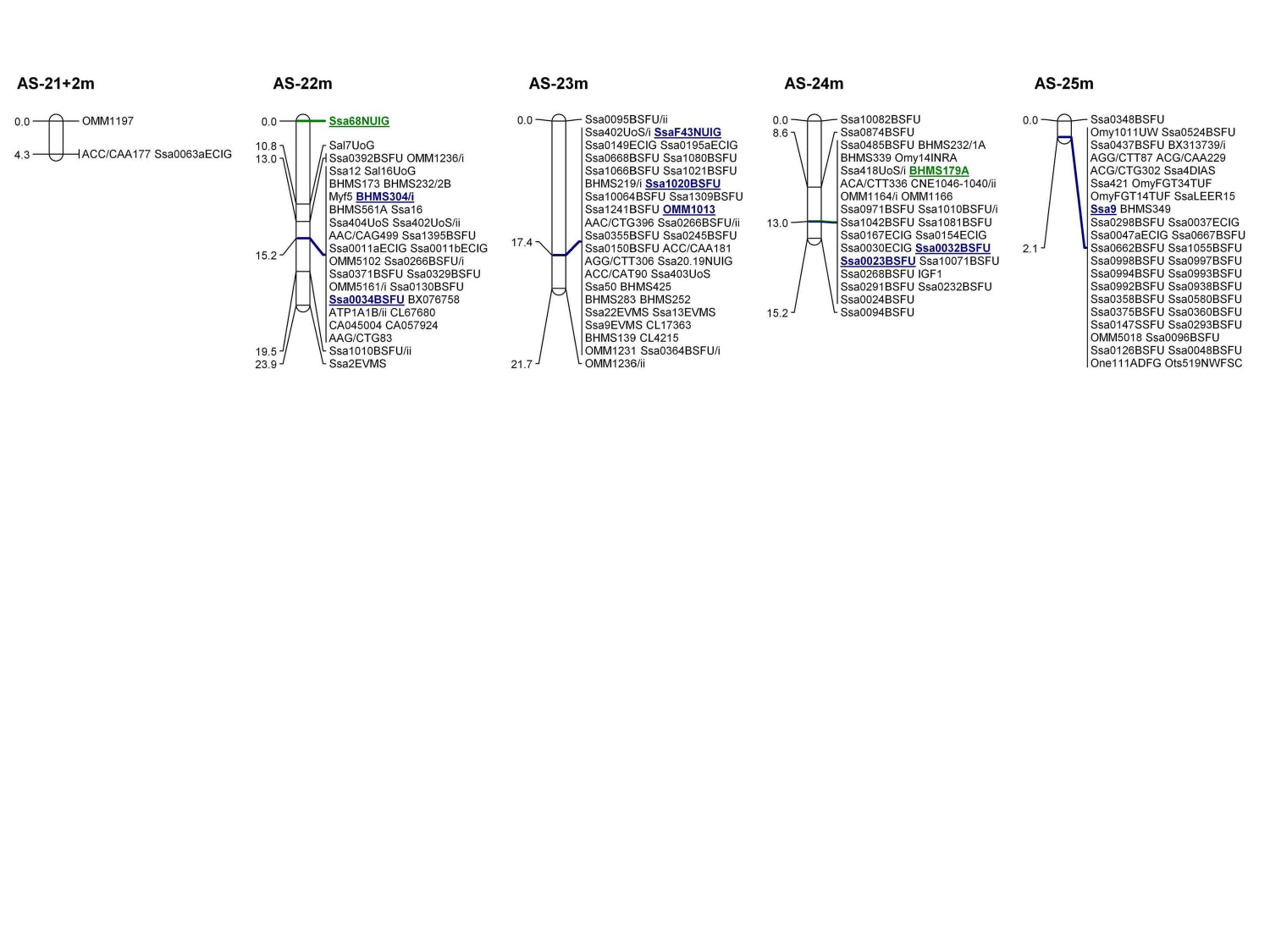

## Slide 7
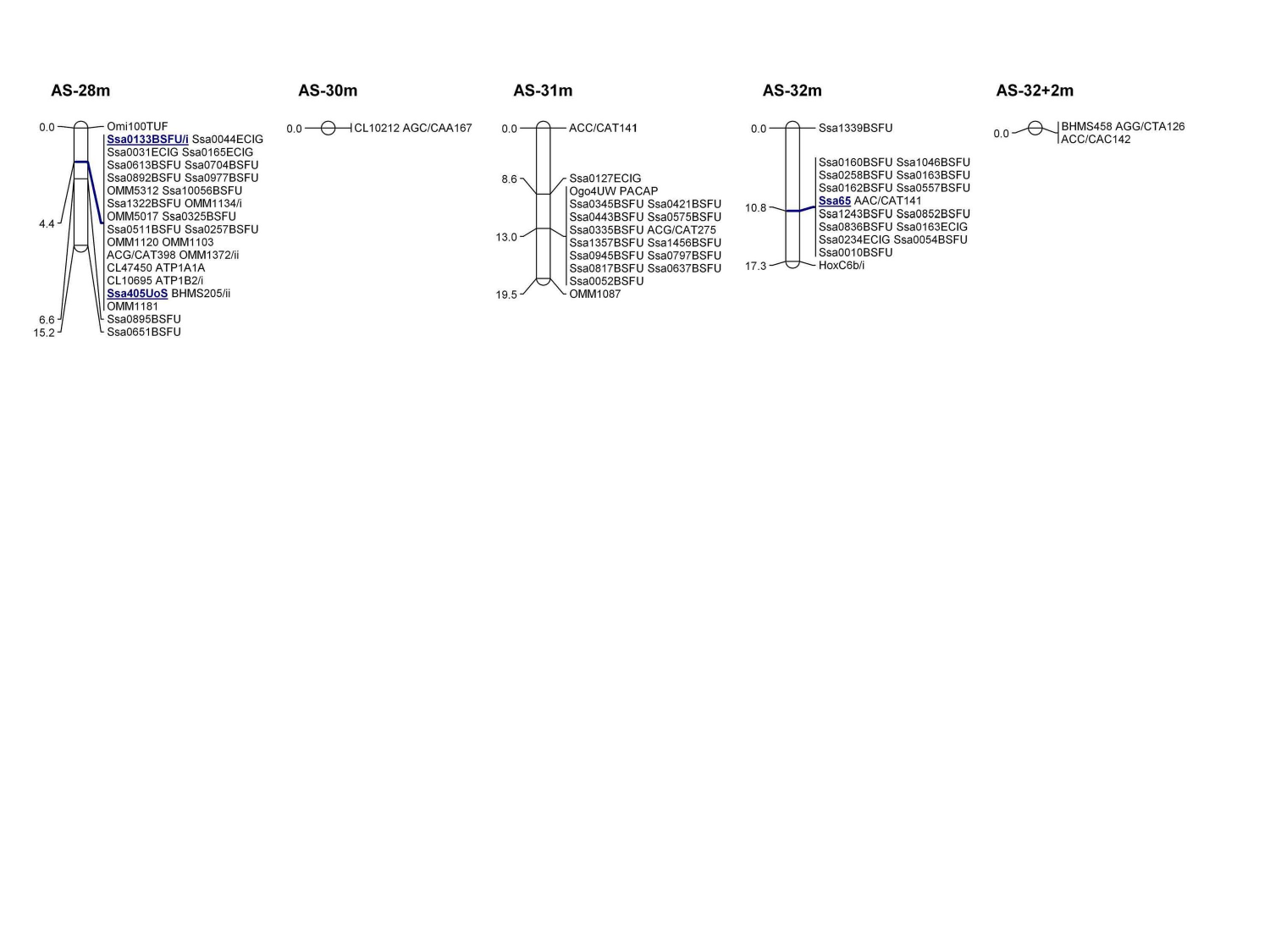

## Slide 8
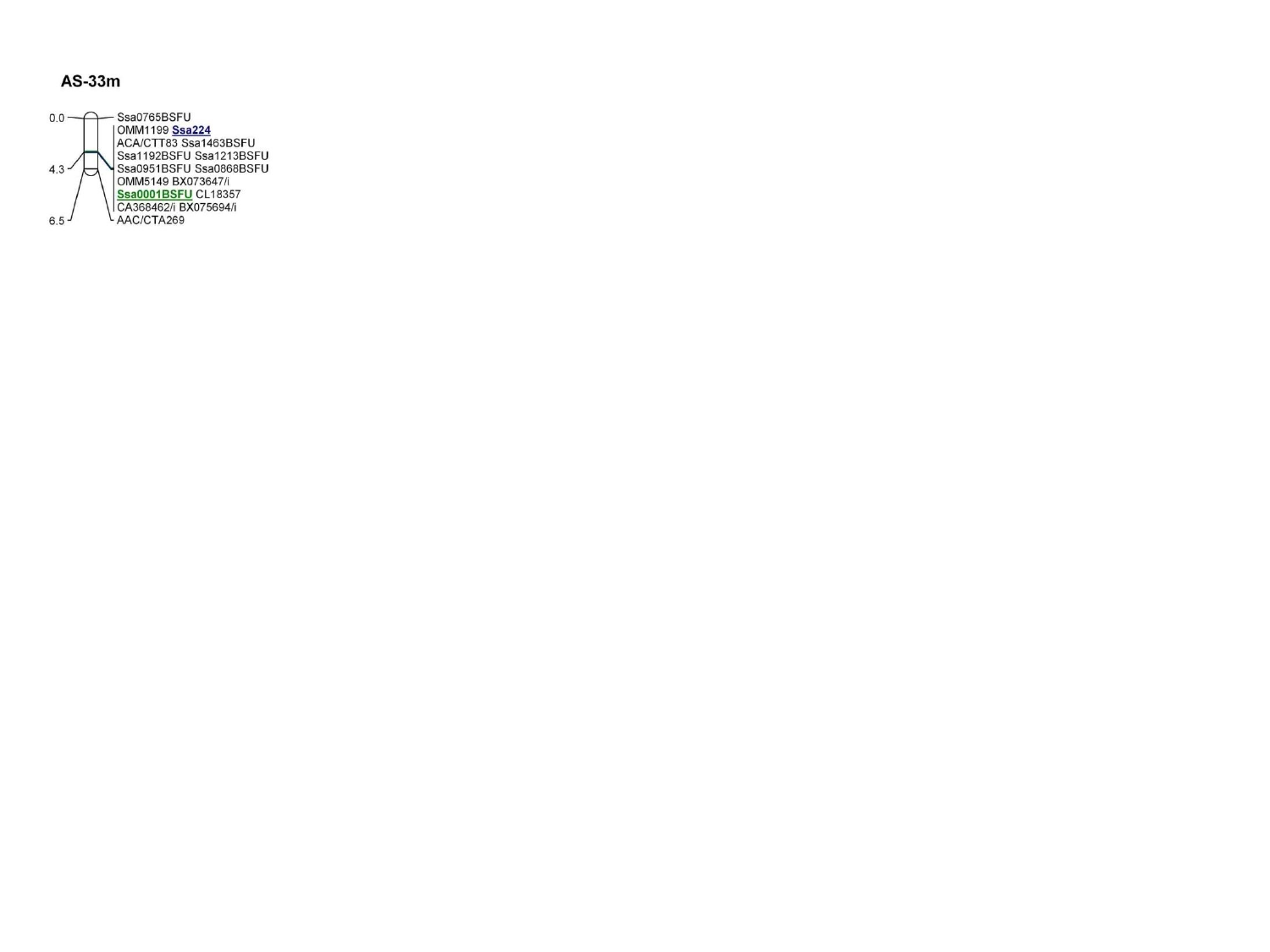

Supplement: Additional file 3 — Figure 2. Atlantic salmon male genetic map from the Br5 family. This figure shows the male genetic map that was constructed based on the SALMAP Atlantic salmon Br5 mapping family. [file 1471-2156-10-46-S3.ppt]

## Slide 1
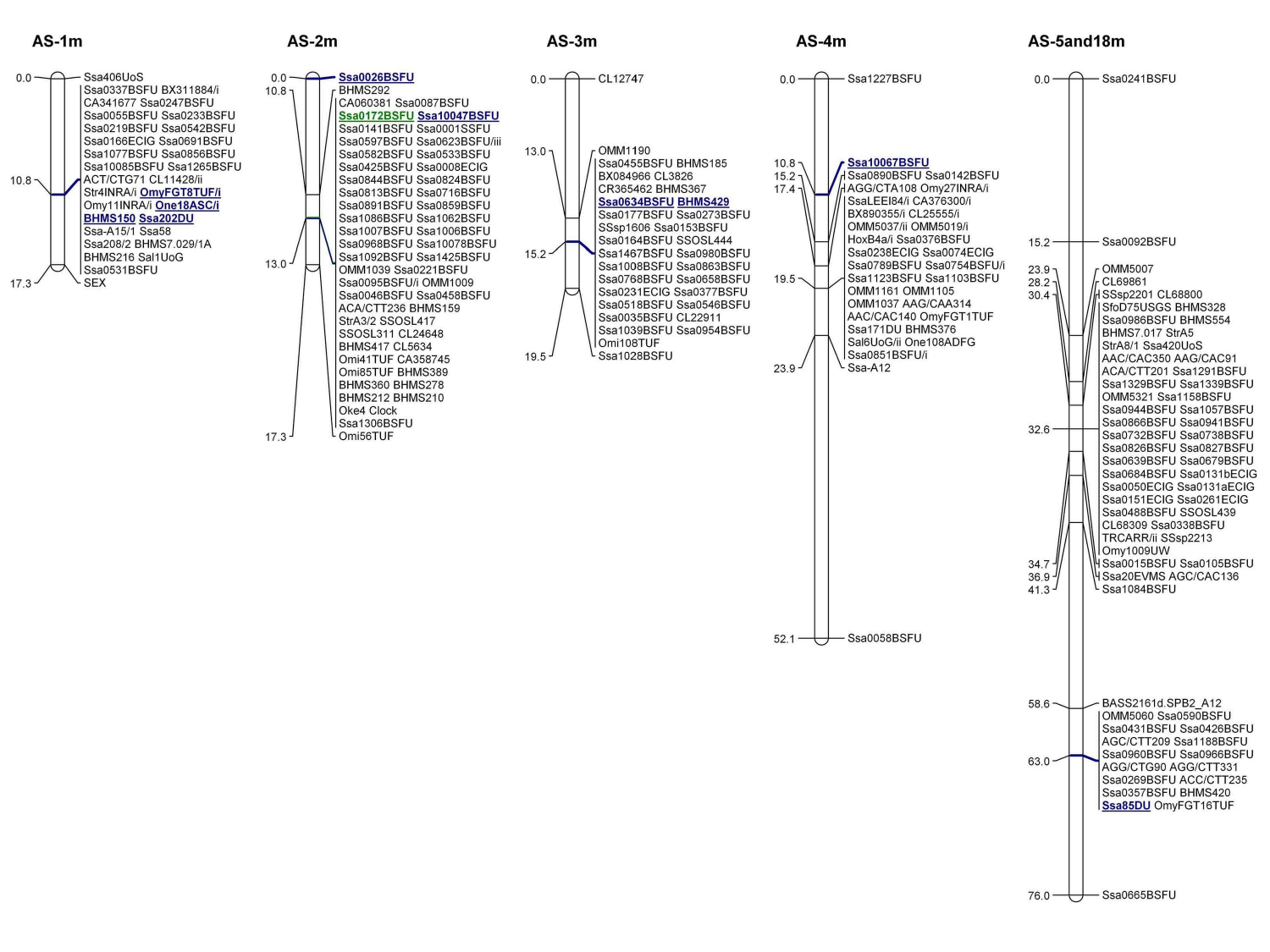

## Slide 2
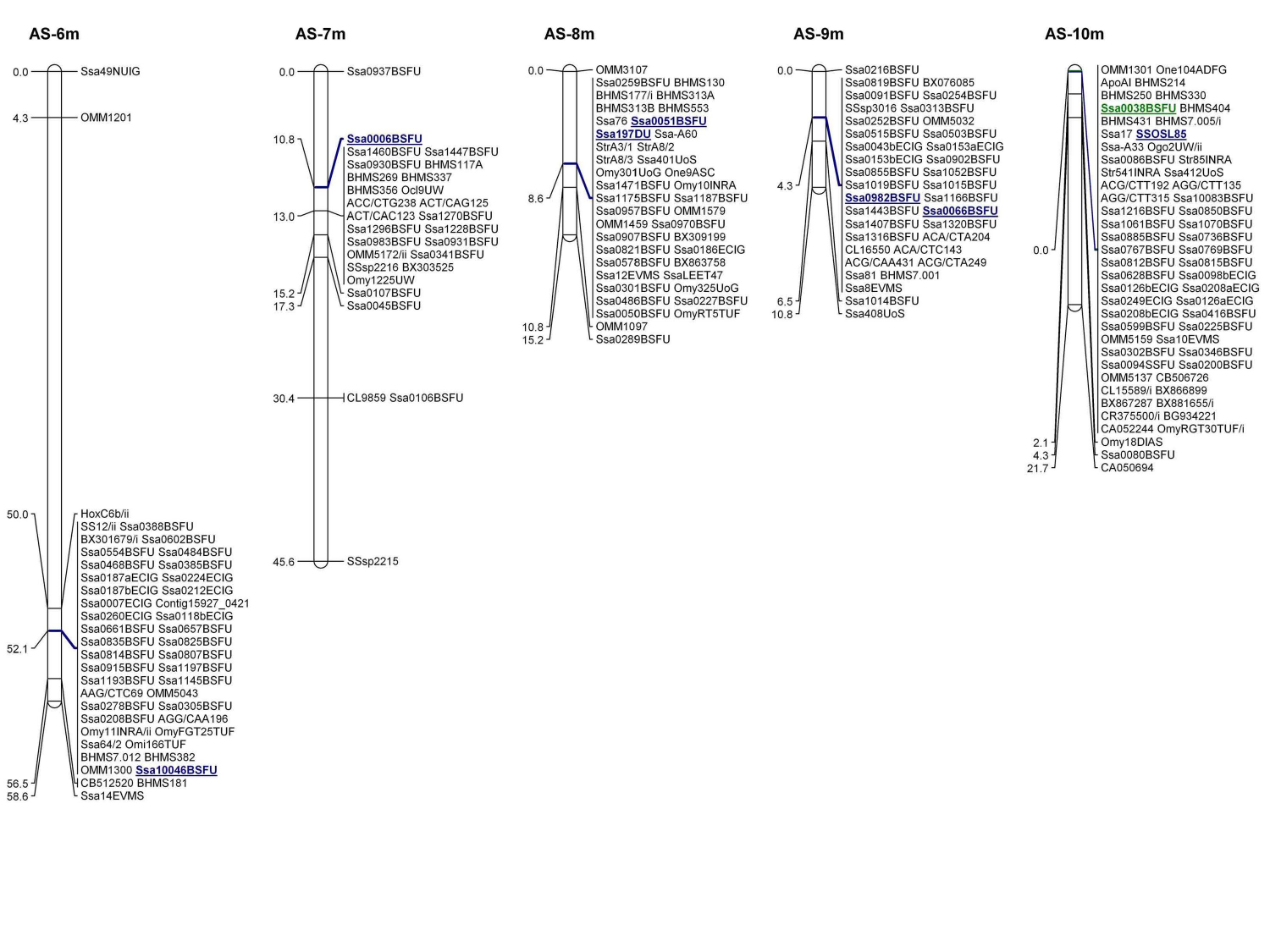

## Slide 3
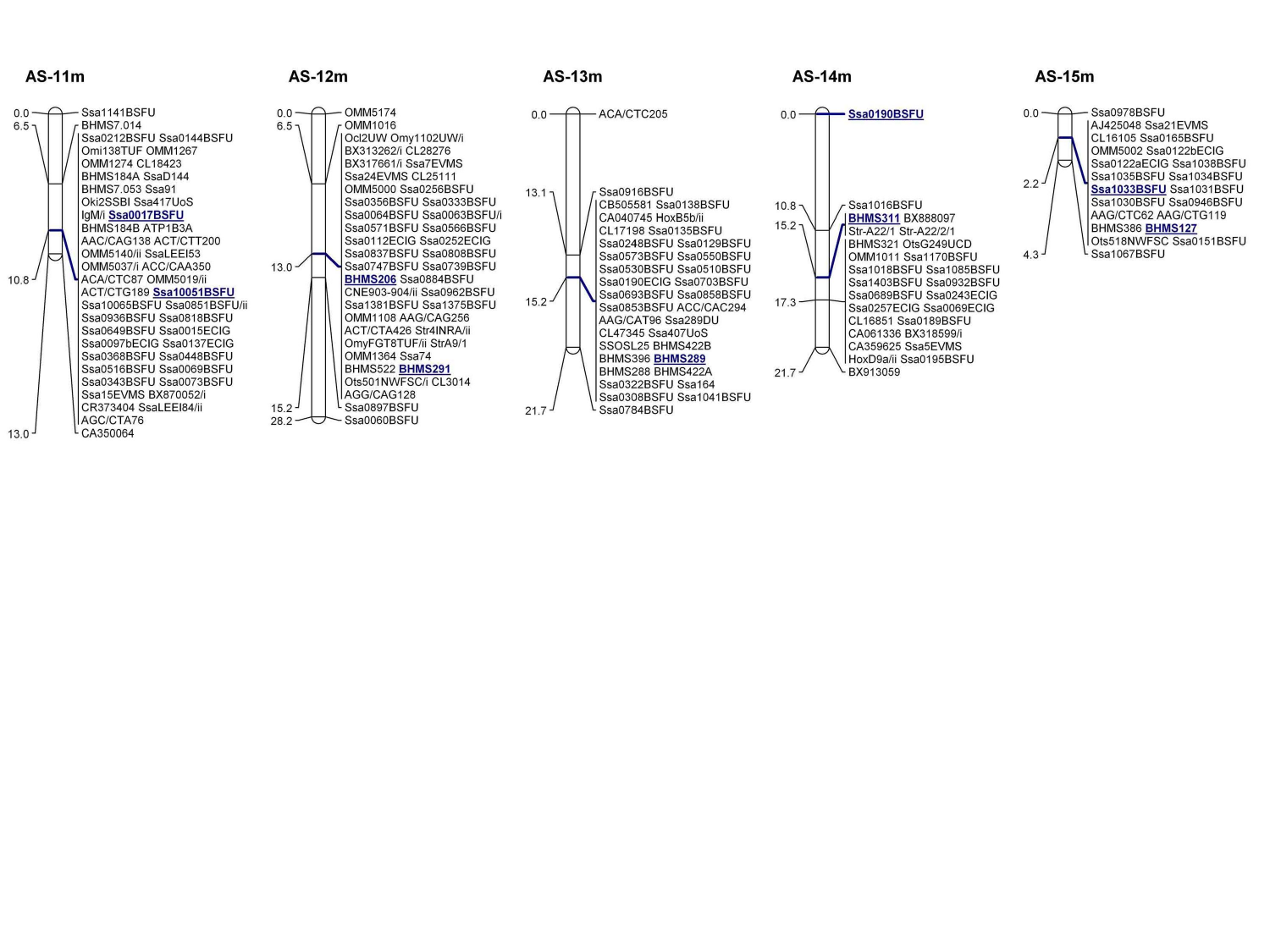

## Slide 4
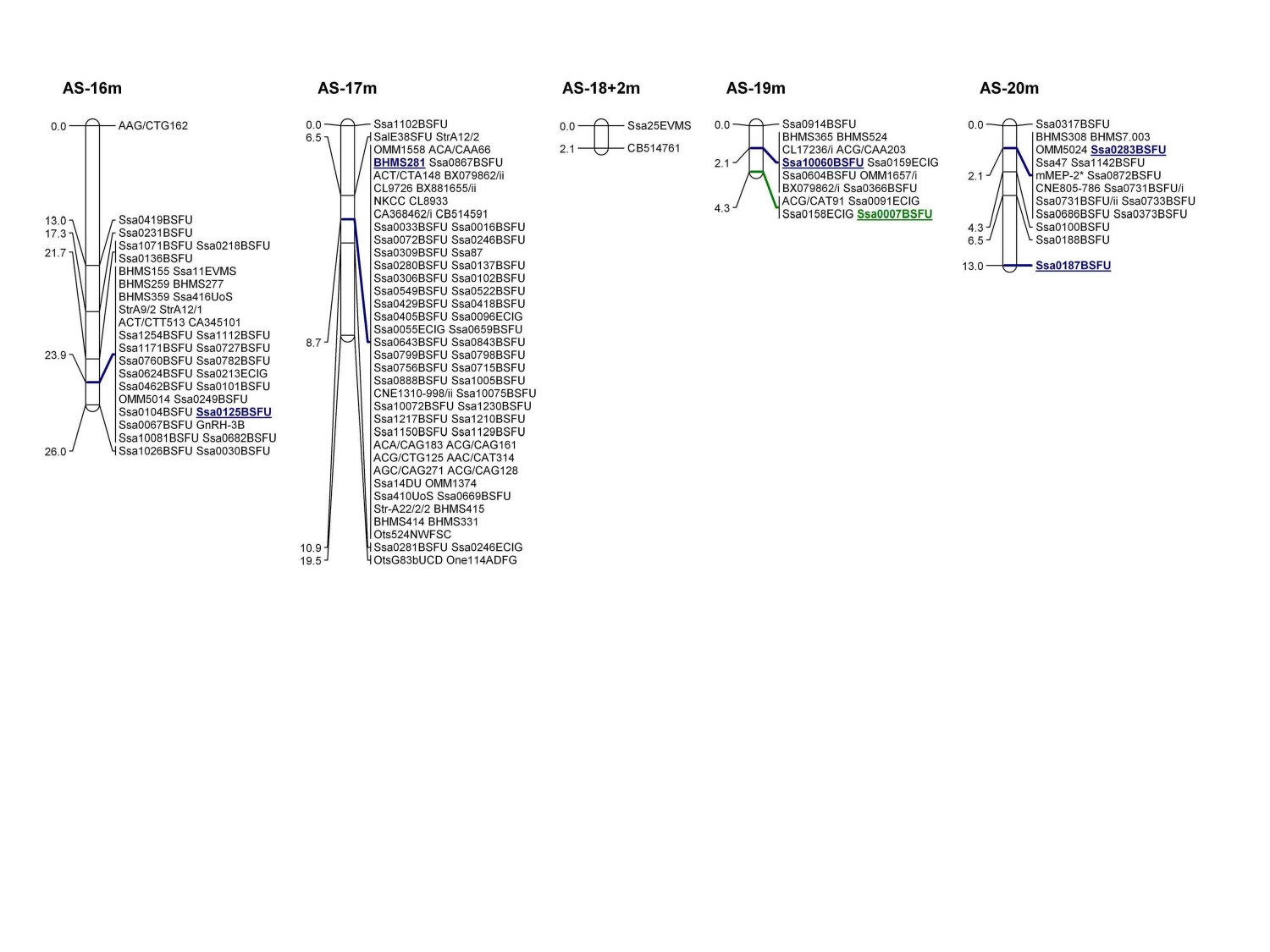

## Slide 5
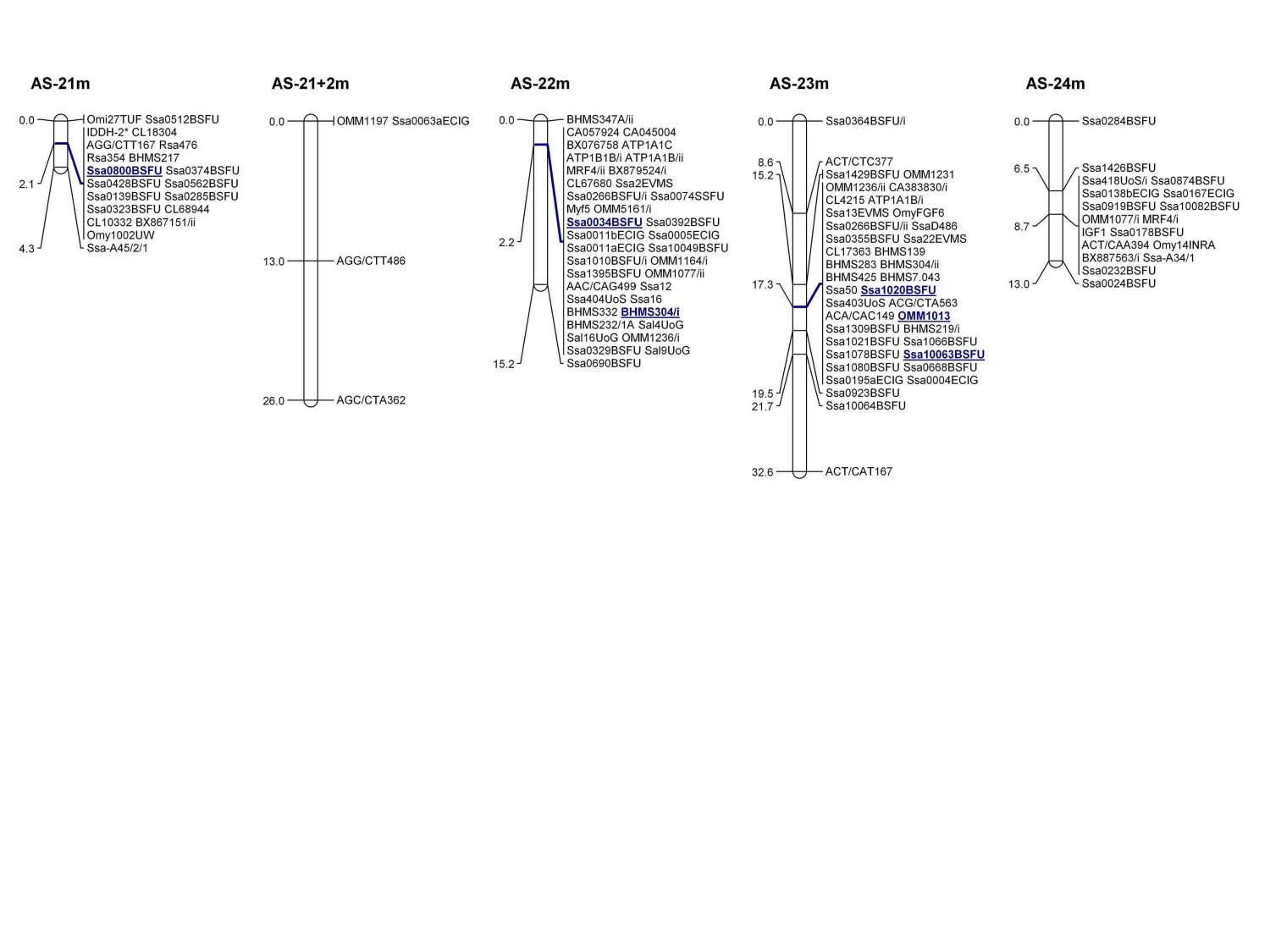

## Slide 6
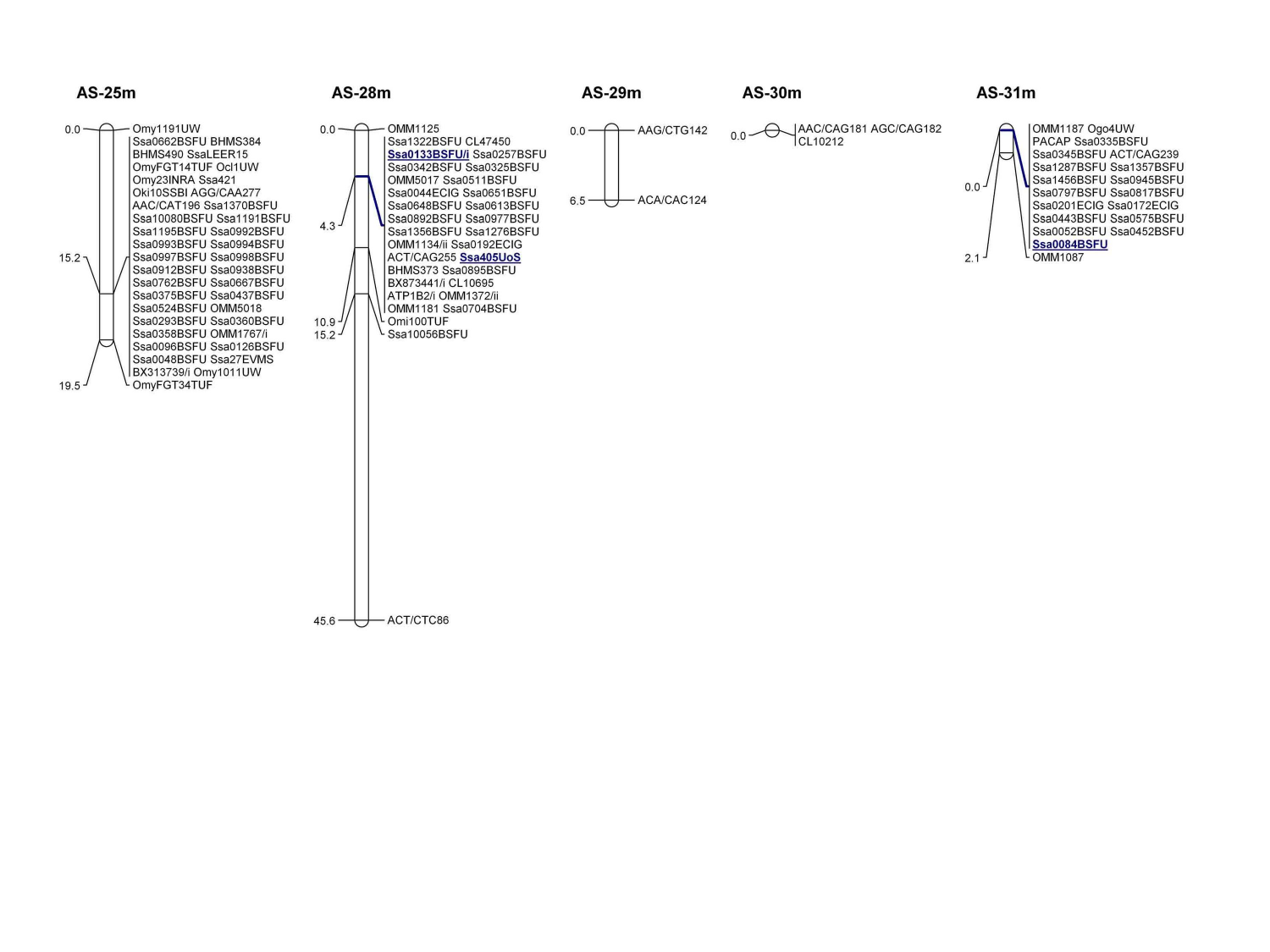

## Slide 7
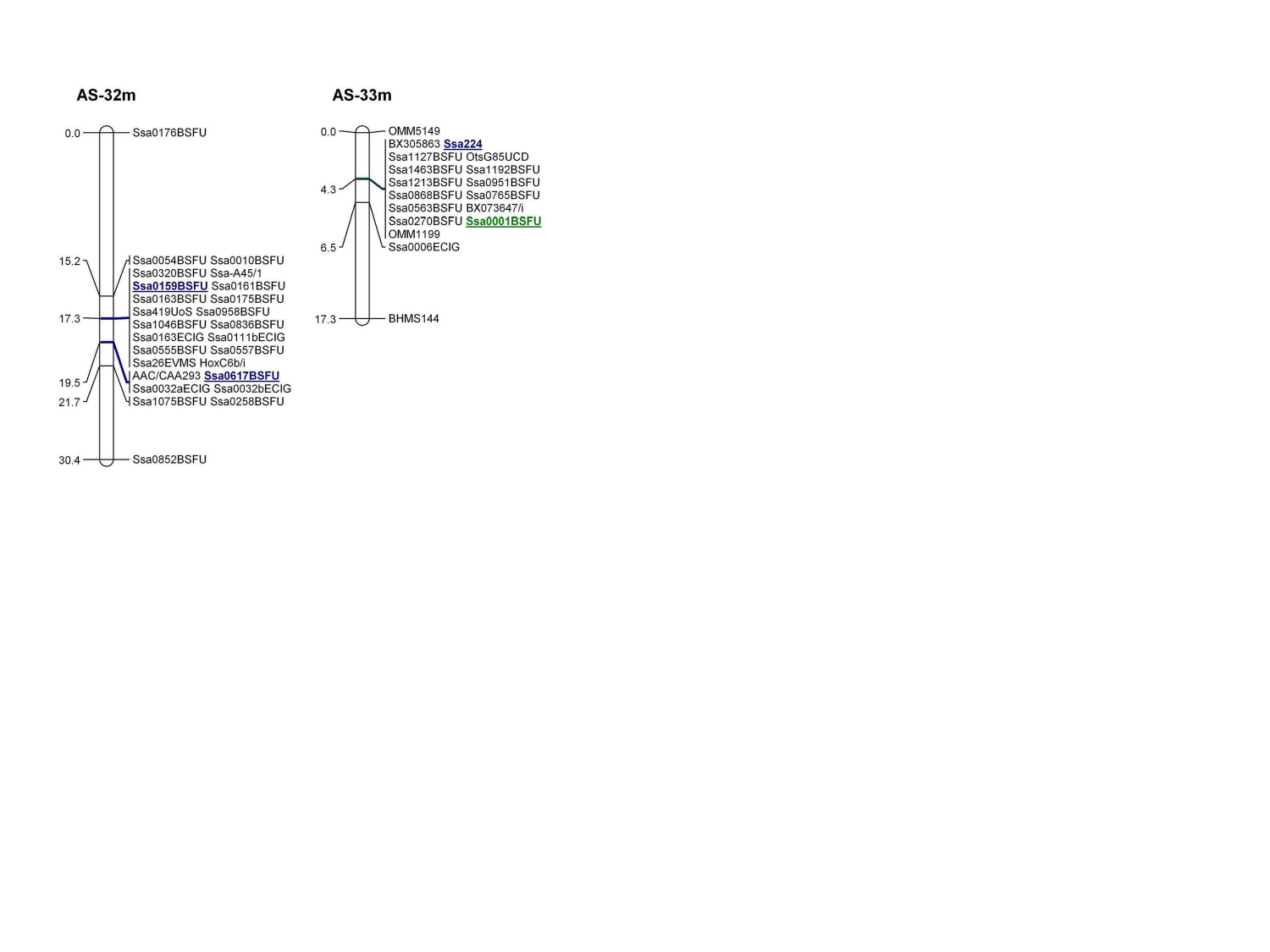

Supplement: Additional file 4 — Figure 3. Atlantic salmon male genetic map from the Br6 family. This figure shows the male genetic map that was constructed based on the SALMAP Atlantic salmon Br6 mapping family. [file 1471-2156-10-46-S4.ppt]
